# Supplementary material for: Myeloid NGS Analyses of Paired Samples from Bone Marrow and Peripheral Blood Yield Concordant Results: A Prospective Cohort Analysis of the AGMT Study Group
Source: Cancers (Basel). 2023 Apr 14;15(8):2305. doi: 10.3390/cancers15082305 (PMC10136651; doi:10.3390/cancers15082305)
Supplement: Supplementary file 1 [file cancers-15-02305-s001.zip › cancers-2297899-supplementary.pdf]

Table S1. Published articles comparing non-NGS analyses from bone marrow and peripheral blood in patients with MDS, CMML and AML.[1]

First author	Year	Patients, n	Paired-BM PB samples, n	Disease	Method	Company, kit	Days between BM-PB analyses, mean (min-max)	Concordance between paired samples (BM and PB)	Concordance between paired mutations BM and PB	Coefficient
Mohmedali A.M.[46]	2015	201	201	• MDS, 183/201 (91%)<br>• AML, 11/201 (5%)<br>• CMML, 7/201 (3%)	SNP-array	• Affymetrix CytoScan HD Array Kit	Not reported	• Complete concordance: 190/201 (95%)<br>• Partial concordance: 11/201 (5%)	Concordance: 356/396 (89.9%)	Not given
Godwin C.D.[47]	2021	477	724	• AML, 477/477 (100%)	Flow-MRD	Beckman Coulter (Fullerton; CA) or Becton Dickinson (BD Bioscience; San Jose, CA) (27 Antibodies)	Not reported (0-7 days)	• Complete concordance: 686/724 (95%)<br>• Partial concordance: not applicable	Not applicable	r=0.92
Fakhr Z.A.[37]	2018	35	35	• MDS, 35/35 (100%)	Karyotype	Not applicable	Not reported	• Complete concordance: 29/35 (83%)<br>• Partial concordance: not given	Not given	Not given
Fakhr Z.A.[37]	2018	35	35	• MDS, 35/35 (100%)	FISH	Not applicable	Not reported	• Complete concordance: 35/35 (100%)<br>• Partial concordance: not given	Not given	Not given
Braulke F.[38]	2013	360	360	• MDS, 360/360 (100%)	FISH (CD34+)	Not applicable	Not reported	• Complete concordance: not given<br>• Partial concordance: not given	Not given	τ=0.96 p<0.01
Cherry A.M.[39]	2012	100	100	• MDS,	FISH	8 Vysis probe sets, with 8 probes, Abbott Molecular Diagnostics	Not reported (0-3 days)	• Complete concordance: 97/100 (97%)<br>• Partial concordance: not given	Not given	Not given
Coleman JF.[48]	2011	48	48	• MDS, 48/48 (100%)	FISH	Not applicable	Not reported (0-180)	• Complete concordance: 30/48 (63%)<br>• Partial concordance: 5/48 (10%)	Not given	Not given

[1]Search terms used in PubMed from 5th of June 2022 until the 7th of December.2022 were: Concordance peripheral blood bone marrow, NGS peripheral blood bone marrow, Myeloid neoplasm peripheral blood bone marrow.
BM indicates Bone marrow, PB, peripheral blood; MDS, myelodysplastic neoplasia; AML, acute myeloid leukemia; CMML chronic myelomonocytic leukemia; SNP, single nucleotide polymorphism; MRD, minimal residual disease; FISH, fluorescence in situ hybridization.

Table S2. Analyzed genes and translocation partners.

<table>
<tr><th>Method</th><th>Platform</th><th>Granularity</th><th>Genes</th><th>Kit used</th></tr>
<tr><td>Next generation sequencing, targeted sequencing</td><td>NextSeq550, MiSeqDx</td><td>All exons</td><td>ASXL1, BCOR, CALR, CEBPA, ETV6, EZH2, IKZF1, NF1, PHF6, PRPF8, RB1, RUNX1, SH2B3, STAG2, TET2, TP53, ZRSR2</td><td rowspan="2">AmpliSeq™ myeloid panel from Illumina® DNA panel</td></tr>
<tr><td>Next generation sequencing, targeted sequencing</td><td>NextSeq550, MiSeqDx</td><td>Hotspot mutations</td><td>ABL, BRAF, CBL, CSF3R, DNMT3A, FLT3, GATA2, HRAS, IDH1, IDH2, JAK2, KIT, KRAS, MPL, MYD88, NPM1, NRAS, PTPN11, SETBP1, SF3B1, SRSF2, U2AF1 and WT1</td></tr>
<tr><td>Next generation sequencing, targeted sequencing</td><td>NextSeq550, MiSeqDx</td><td>Fusion driver genes (translocation partners)</td><td>ABL1, ALK, BCL2, BRAF, CCND1, CREBBP, EGFR, ETV6, FGFR1, FGFR2, FUS, HMGA2, JAK2, KMT2A (MLL), MECOM, MET, MLLT10, MLLT3, MYBL1, MYH11, NTRK3, NUP214, PDGFRA, PDGFRB, RARA, RBM15, RUNX1, TCF3 and TFE3</td><td>AmpliSeq™ myeloid panel from Illumina® RNA panel</td></tr>
<tr><td>Fragment analysis</td><td>ABI Genetic Analyser 3500</td><td>Hotspots
Internal tandem repeats</td><td>D835 and I836; FLT3 tyrosine kinase domain (TKD)
Juxtamembrane (JM) region; FLT3 internal tandem repeats (ITD)</td><td>LeukoStrat® FLT3 Mutation Assay 2.0 from Invivoscribe</td></tr>
</table>

Modified according to https://science-docs.illumina.com/documents/LibraryPrep/ampliseq-myeloid-panel-data-sheet-770-2018-016/Content/Source/Library-Prep/AmpliSeq/myeloid-panel/ampliseq-myeloid-panel-data-sheet.html

Figure S1. Occurrence of mutations by gene and by sample type for all samples of patients with AML (n=46 patients, n= 57 sample pairs[1,2], n=396 mutations detected[3,4]).

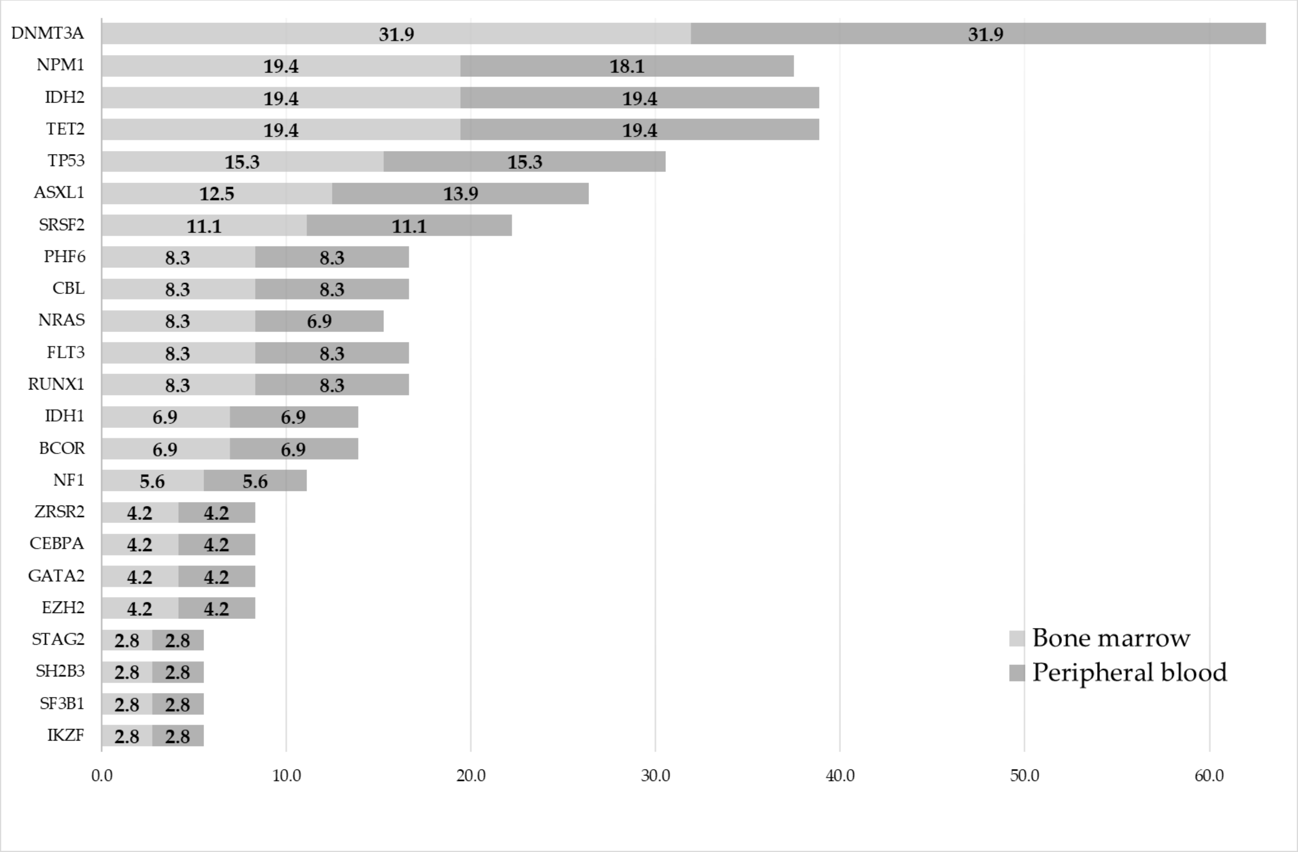

The X-axis represents the percentage of occurrence of mutations in bone marrow and peripheral blood samples.
The mean VAF of all 396 detected mutations in bone marrow was 23.4% (SD 15.1) and 20.9% (SD 14.9) in the peripheral blood (spearman correlation coefficient 0.93, p= 0.001).

[1]Includes 26 serial sample pairs from 12 patients.
[2]Total sample pairs (n=72), excluding sample pairs without mutations (n=15), and excluding sample pairs in which only fusion genes (and no other mutations) were found (n=0).
[3]Mutations occurring in <2% of the AML cohort were not included in the graph and included following genes for bone marrow vs peripheral blood, respectively: KIT, BRAF, ETV6, HRAS, JAK2, MPL, PRPF8, PTPN11 and WT1 with 1.4% vs 1.4% each.
[4]If a sample had more than one mutation in the same gene, it was only counted once.

Figure S2. Occurrence of mutations by gene and by sample type for all samples of patients with MDS (n=43 patients, n= 55 sample pairs[1,2], n=405 mutations detected[3,4]).

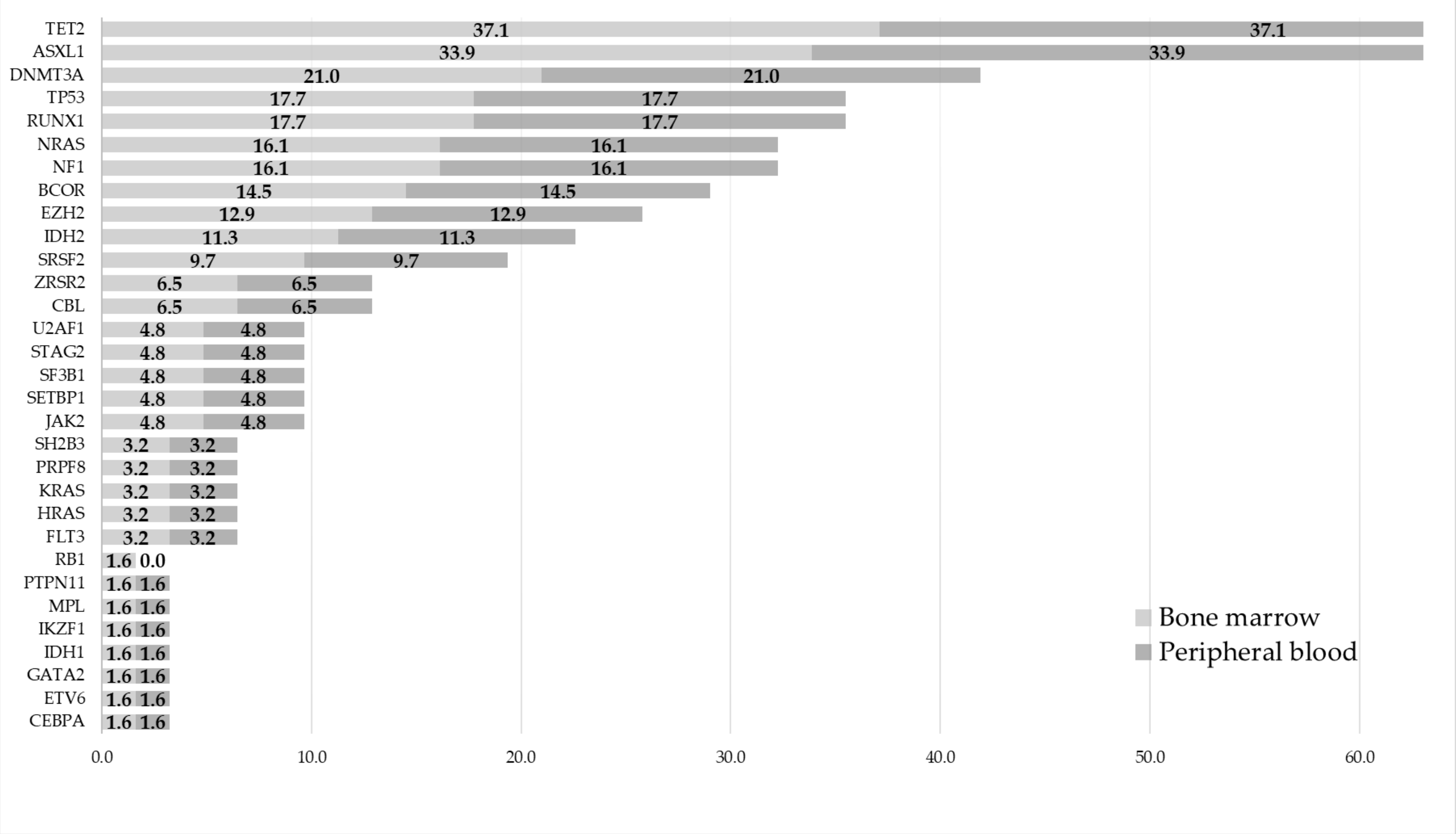

The X-axis represents the percentage of occurrence of mutations in bone marrow and peripheral blood samples.
The mean VAF of all 405 detected mutations in bone marrow was 24.1% (SD 16.3) and 21.7% (SD 15.1) in the peripheral blood (spearman correlation coefficient 0.98, p= 0.001).

[1]Includes 28 serial sample pairs from 10 patients.
[2]Total sample pairs (n=63), excluding sample pairs without mutations (n=7), and excluding sample pairs in which only fusion genes (and no other mutations) were found (n=1).
[3]There were no mutations occurring <1.6%.
[4]If a sample had more than one mutation in the same gene, it was only counted once.

Figure S3. Occurrence of mutations by gene and by sample type for all samples of patients with MDS-MPN (n=15 patients, n= 15 sample pairs[1,2], n=138 mutations detected[3,4]).

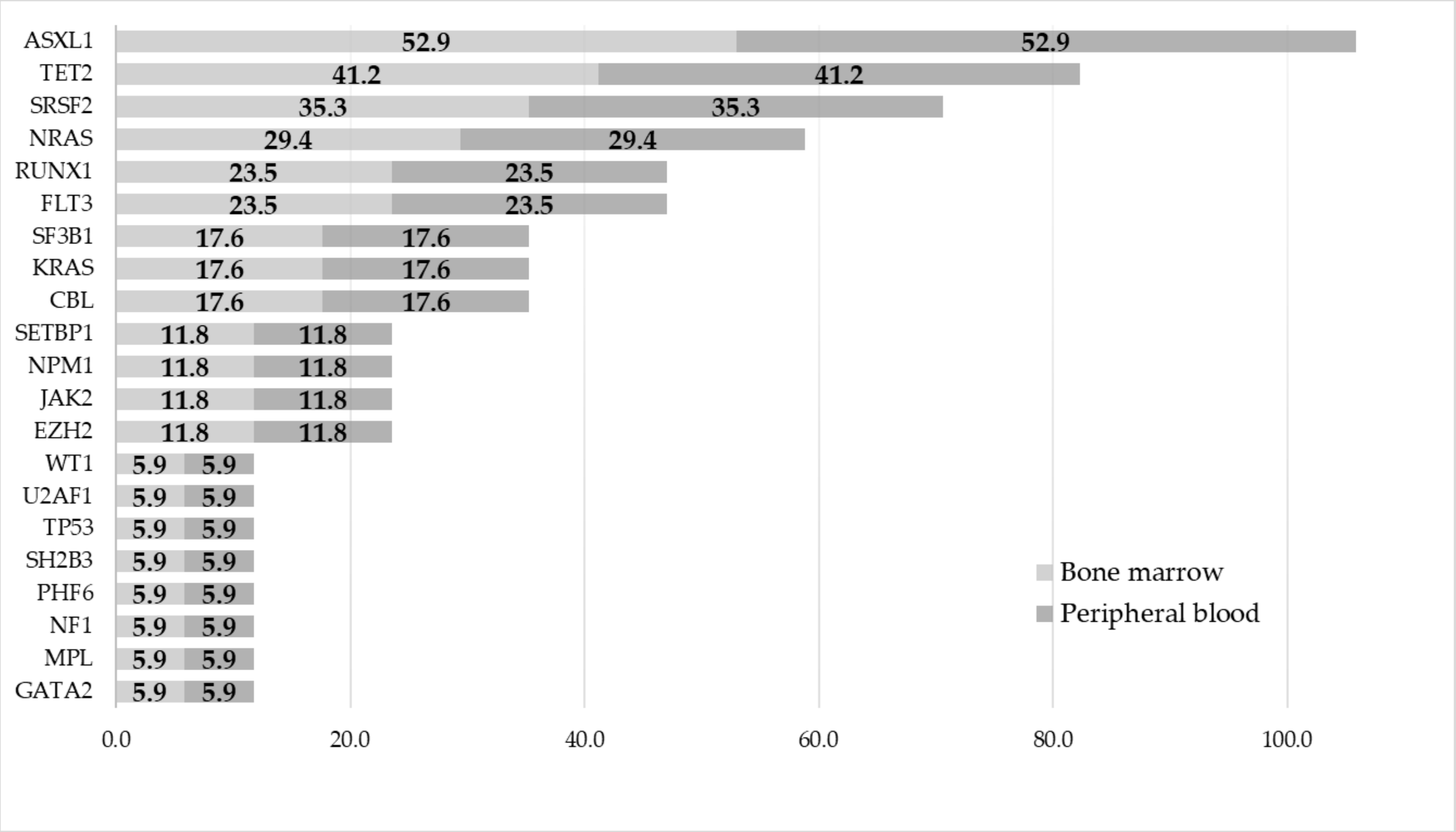

The X-axis represents the percentage of occurrence of mutations in bone marrow and peripheral blood samples.
The mean VAF of all 138 detected mutations in bone marrow was 31.0% (SD 23.0) and 28.7% (SD 23.2) in the peripheral blood (spearman correlation coefficient 0.97, p= 0.007).

[1]Includes 2 serial sample pair from one patient.
[2]Total sample pairs (n=16), excluding sample pairs without mutations (n=1), and excluding sample pairs in which only fusion genes (and no other mutations) were found (n=0).
[3]There were no mutations occurring <5.9%.
[4]If a sample had more than one mutation in the same gene, it was only counted once.

Figure S4. Occurrence of mutations by gene and by sample type for all samples of patients with MPN[1] (n=33 patients, n= 33 sample pairs[2,3], n=235 mutations detected[4,5]).

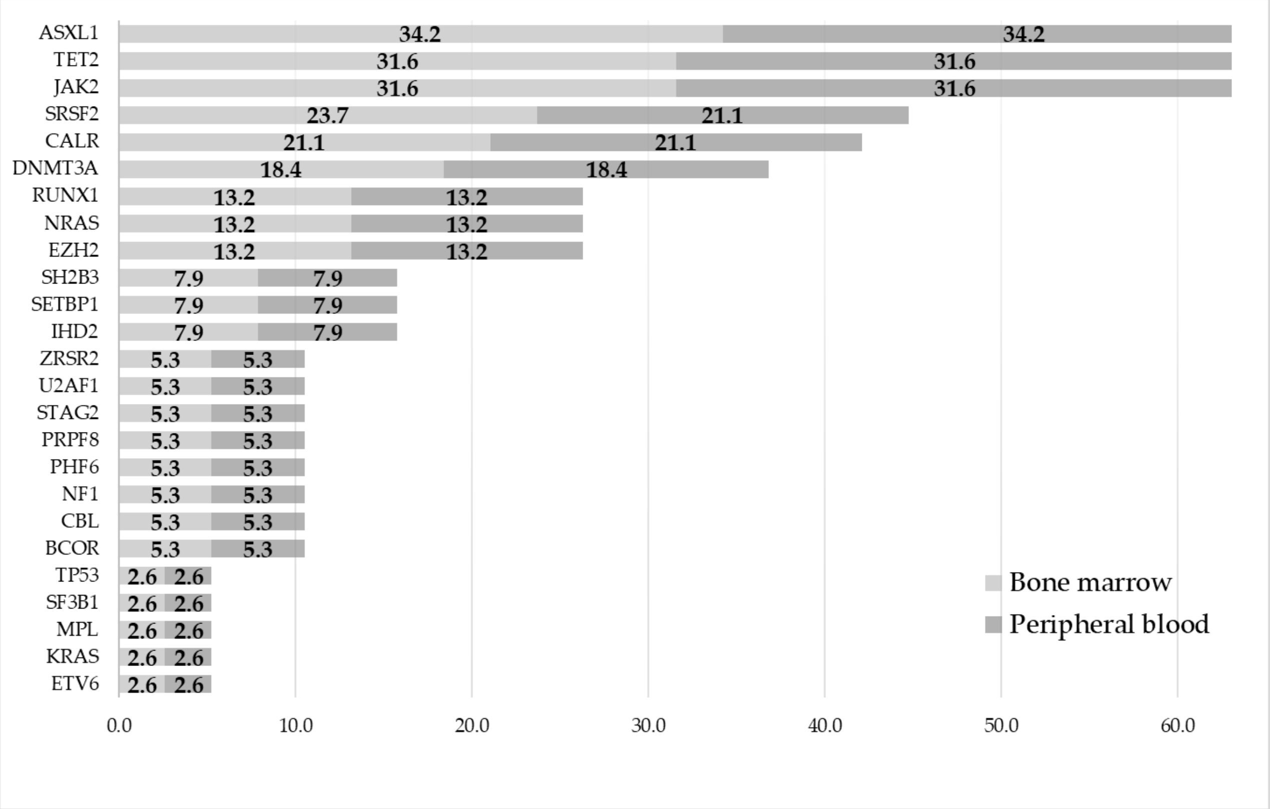

The X-axis represents the percentage of occurrence of mutations in bone marrow and peripheral blood samples.
The mean VAF of all 235 detected gene mutations in bone marrow was 26.6% (SD 16.2) and 24.9% (SD 16.0) in the peripheral blood (spearman correlation coefficient 0.98, p= 0.013).

[1]Includes the following diagnoses: chronic myelogenous leukemia, essential thrombocytosis, polycythemia vera, primary myelofibrosis, mastocytosis, idiopathic hypereosinophilic syndrome.
[2]Includes 7 serial sample pairs from 2 patients.
[3]Total sample pairs (n=38), excluding sample pairs without mutations (n=2), and excluding sample pairs in which only fusion genes (and no other mutations) were found (n=3).
[4]There were no mutations occurring <2.6%.
[5]If a sample had more than one mutation in the same gene, it was only counted once.

Figure S5. Occurrence of mutations by gene and by sample type for all samples of patients with non-myeloid diagnoses[1] (n=50 patients, n= 31 sample pairs[2,3], n=119 mutations detected[4,5]).

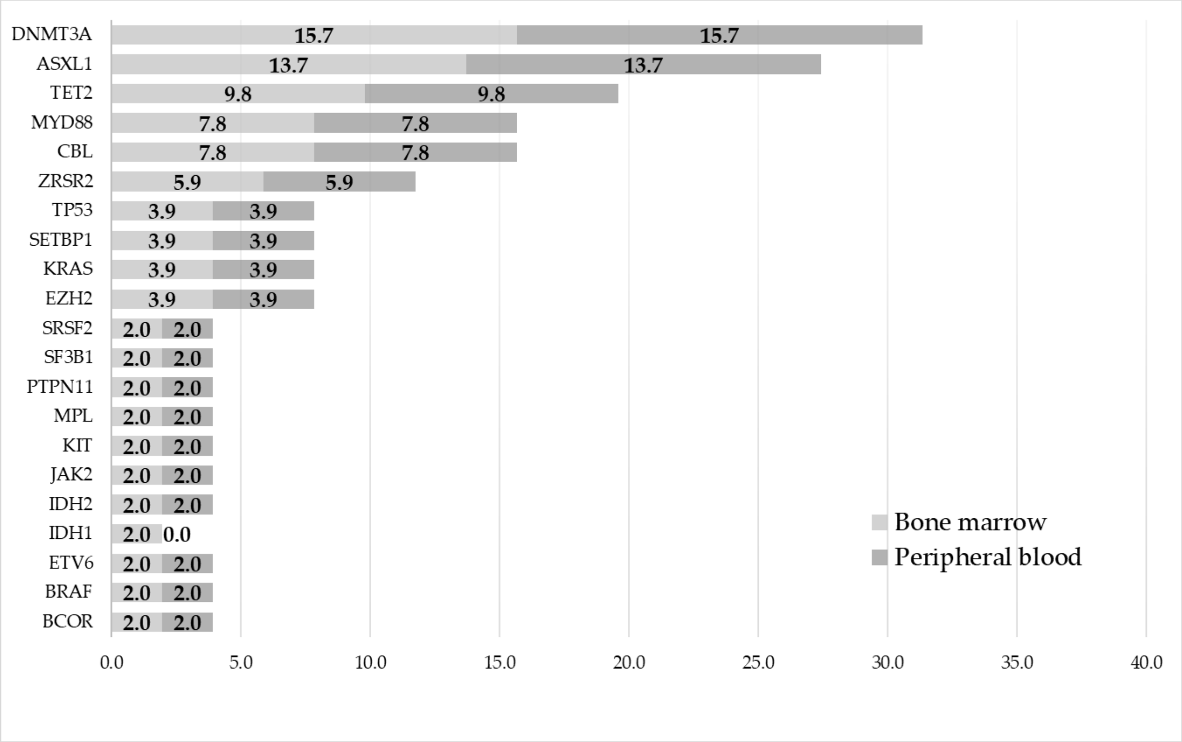

The X-axis represents the percentage of occurrence of mutations in bone marrow and peripheral blood samples.
The mean VAF of all 119 detected mutations in bone marrow was 26.1% (SD 25.0) and 22.9% (SD 23.2) in the peripheral blood (spearman correlation coefficient 0.92, p= 0.013).

[1]Includes patients with cytopenias and/or cytoses with the following diagnoses: B-cell lymphomas (n=10), myeloma (n=7), solid tumors (n=7), rheumatic diseases (n=3), pernicious anemia (n=3), reactive cytoses (n=3), aplastic anemia (n=2), drug induced bone marrow toxicity (n=2), autoimmune hemolytic anemia/kryoglobulinemia (n=2), liver cirrhosis (n=2), familial Mediterranean fever (n=1), human immunodeficiency virus infection (n=1), hypereosinophilic syndrome (n=1), cutaneous mastocytosis (n=1), and unexplained cytopenias (n=5).
[2]Includes 1 serial sample pair from 1 patient.
[3]Total sample pairs (n=51), excluding sample pairs without mutations (n=20), and excluding sample pairs in which only fusion genes (and no other mutations) were found (n=0).
[4]There were no mutations occurring <2%.
[5]If a sample had more than one mutation in the same gene, it was only counted once.

Figure S6. Mean variant allele frequency (VAF) by gene and by sample type for all samples of all patients (n=150 patients, n= 191 sample pairs[1,2]**,** n=1289 gene mutations detected[3]).

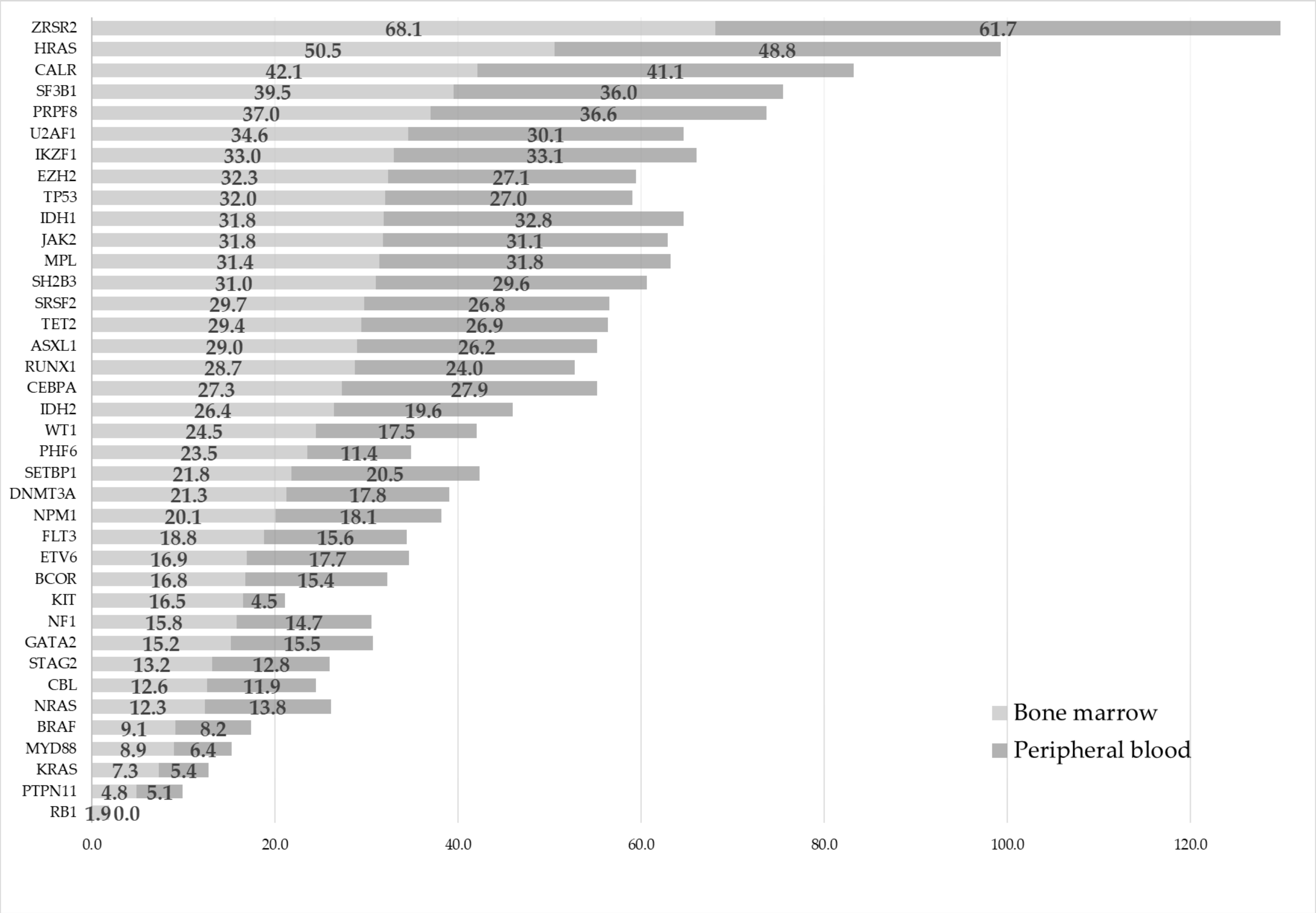

The X-axis represents the percentage of VAFs of the detected mutations in bone marrow and peripheral blood samples.

[1]Includes 53 serial sample pairs from 30 patients.
[2]Total sample pairs (n=240), excluding sample pairs without mutations (n=46), and excluding sample pairs in which only fusion genes (and no other mutations) were found (n=3).
[3]FLT3-ITD mutations are not included in the diagram as the method used for detection does not produce VAF values.

Table S3. Mean variant allele frequency (VAF) by gene and by sample type and diagnosis.

Total cohort			MDS			AML			MDS/MPN			MPN			Others		
Gene	Mean BM_{VAF}, %	Mean PB_{VAF}, %	Gene	Mean BM_{VAF}, %	Mean PB_{VAF}, %	Gene	Mean BM_{VAF}, %	Mean PB_{VAF}, %	Gene	Mean BM_{VAF}, %	Mean PB_{VAF}, %	Gene	Mean BM_{VAF}, %	Mean PB_{VAF}, %	Gene	Mean BM_{VAF}, %	Mean PB_{VAF}, %
ZRSR2	68.1	61.7	ZRSR2	55.2	48.6	ZRSR2	59.5	57.2	MPL	96.7	96.9	ZRSR2	64.0	64.6	ZRSR2	92.5	81.7
HRAS	50.5	48.8	IKZF1	50.3	49.2	HRAS	51.6	50.3	EZH2	63.9	59.5	PRPF8	49.8	49.9	EZH2	64.9	48.8
CALR	42.1	41.1	HRAS	50.0	48.0	SH2B3	46.1	46.5	TET2	54.8	52.9	U2AF1	45.0	45.2	JAK2	50.8	50.5
SF3B1	39.5	36.0	ETV6	47.3	49.2	IDH1	41.6	35.4	SH2B3	47.8	48.5	SF3B1	44.2	37.9	MPL	49.8	50.1
PRPF8	37.0	36.6	IDH1	43.9	34.3	TP53	36.7	32.4	SF3B1	45.4	42.4	CALR	42.1	41.1	SETBP1	45.7	37.4
U2AF1	34.6	30.1	PRPF8	42.2	41.4	CEBPA	35.1	35.9	ASXL1	41.0	39.2	JAK2	39.0	38.0	IDH2	43.3	41.6
IKZF1	33.0	33.1	IDH2	38.7	29.4	PHF6	34.2	16.3	TP53	40.8	44.3	TET2	37.3	35.3	SRSF2	41.8	40.9
EZH2	32.3	27.1	SF3B1	36.5	29.8	SF3B1	32.9	33.0	U2AF1	40.8	32.5	RUNX1	36.8	34.5	SF3B1	39.8	39.3
TP53	32.0	27.0	SRSF2	32.4	27.8	BCOR	31.6	30.1	WT1	33.7	26.2	TP53	36.3	21.0	ASXL1	26.0	23.6
IDH1	31.8	32.8	DNMT3A	31.1	24.1	SRSF2	31.0	25.9	RUNX1	28.7	25.6	STAG2	29.5	29.1	BRAF	16.0	13.4
JAK2	31.8	31.1	TP53	29.8	24.0	NF1	30.7	25.4	SRSF2	28.2	25.1	ASXL1	27.5	26.6	TP53	12.8	5.3
MPL	31.4	31.8	EZH2	29.6	26.0	TET2	29.3	26.0	SETBP1	25.2	22.0	NRAS	27.3	24.2	TET2	11.7	10.3
SH2B3	31.0	29.6	FLT3	29.1	27.9	RUNX1	28.2	18.1	CBL	18.5	14.3	SRSF2	26.5	26.4	KIT	9.8	1.1
SRSF2	29.7	26.8	ASXL1	27.4	24.5	ASXL1	26.9	18.3	FLT3	18.3	13.9	BCOR	24.4	24.8	ETV6	9.2	20.2
TET2	29.4	26.9	SH2B3	25.9	26.0	GATA2	26.3	23.2	JAK2	18.1	17.9	EZH2	23.9	20.4	IDH1	9.1	0.0
ASXL1	29.0	26.2	CBL	25.6	25.3	IKZF	24.3	25.1	NRAS	14.8	15.3	NF1	22.2	21.2	MYD88	8.9	6.4
RUNX1	28.7	24.0	U2AF1	25.5	19.2	DNMT3A	23.8	20.5	KRAS	12.0	9.2	SETBP1	20.2	18.2	DNMT3A	4.5	4.1
CEBPA	27.3	27.9	RUNX1	23.5	20.9	NPM1	23.3	21.8	NPM1	8.1	0.8	SH2B3	18.7	14.5	CBL	4.3	5.1
IDH2	26.4	19.6	TET2	22.7	19.7	IDH2	21.3	14.4	GATA2	7.4	11.8	DNMT3A	16.8	16.3	KRAS	3.6	1.3
WT1	24.5	17.5	JAK2	15.5	15.5	STAG2	19.9	21.5	PHF6	3.5	1.3	IHD2	15.9	13.2	BCOR	1.7	0.4
PHF6	23.5	11.4	NRAS	12.4	11.9	EZH2	18.6	12.9	NF1	2.4	4.2	MPL	9.4	9.9	PTPN11	1.4	0.2
SETBP1	21.8	20.5	SETBP1	11.2	12.7	KIT	17.9	5.2				CBL	5.1	4.4			
DNMT3A	21.3	17.8	NF1	10.1	10.4	FLT3	15.1	12.0				KRAS	2.0	2.4			
NPM1	20.1	18.1	BCOR	7.9	6.3	CBL	10.3	10.0				PHF6	1.3	1.5			
FLT3	18.8	15.6	KRAS	6.6	5.4	ETV6	10.2	0.5				ETV6	0.9	1.1			
ETV6	16.9	17.7	PTPN11	5.8	5.4	WT1	6.1	0.2									
BCOR	16.8	15.4	STAG2	3.9	2.9	PTPN11	5.6	8.9									
KIT	16.5	4.5	CEBPA	3.9	4.1	NRAS	4.0	9.3									
NF1	15.8	14.7	RB1	1.9	0.0	JAK2	2.8	2.8									
GATA2	15.2	15.5	GATA2	1.4	1.3	BRAF	2.23	3.13									
STAG2	13.2	12.8	MPL	0.9	1.1	PRPF8	1.0	0.6									
CBL	12.6	11.9				MPL	0.3	1.2									
NRAS	12.3	13.8															
BRAF	9.1	8.2															
MYD88	8.9	6.4															
KRAS	7.3	5.4															
PTPN11	4.8	5.1															
RB1	1.9	0.0															

BM indicates Bone marrow; VAF, Variant allele frequency; PB peripheral blood.

Table S4. Complete list of reported variants and the laboratory values in the 9 discordant patients.

ID	Serial samples	Analysis	BM blasts, %	PB blasts, %	WBC, G/L	Detected mutation	HGVSp	Pathway	BM_{VAF}, %	PB_{VAF}, %
1	yes	1st	95	8	32.7	FLT3-ITD	Not given[1]	Receptor tyrosine kinase	Not given[1]	Not given[1]
1	yes	1st	95	8	32.7	DNMT3A	p.Arg882His	DNA methylation related	46.9	43.7
1	yes	1st	95	8	32.7	NPM1	p.Trp288Cysfs*12	Nucleolar multifunctional protein	46.5	39.8
1	yes	2nd	2	Not done	0.7	DNMT3A	p.Arg882His	DNA methylation related	31.4	19.2
1	yes	2nd	2	Not done	0.7	NPM1	p.Trp288Cysfs*12	Nucleolar multifunctional protein	9.7	1.8
1	yes	3rd	1	0	4.6	DNMT3A	p.Arg882His	DNA methylation related	43.4	41.9
1	yes	3rd	1	0	4.6	NPM1	p.Trp288Cysfs*12	Diverse cellular processes	0.6	0.0
1	yes	4th	78	0	1.1	DNMT3A	p.Arg882His	DNA methylation related	43.8	39.4
1	yes	4th	78	0	1.1	IDH2	p.Arg140Gln	DNA methylation related	32.1	1.1
1	yes	4th	78	0	1.1	NPM1	p.Trp288Cysfs*12	Nucleolar multifunctional protein	30.0	0.7
1	yes	4th	78	0	1.1	MPL	p.His624Asp	Thrombopoietin receptor	0.3	1.2
1	yes	4th	78	0	1.1	FLT3-ITD	Not given[1]	Receptor tyrosine kinase	Not given[1]	Not given[1]
1	yes	5th	1	0	0.5	DNMT3A	p.Arg882His	DNA methylation related	39.7	23.4
1	yes	5th	1	0	0.5	IDH2	p.Arg140Gln	DNA methylation related	24.7	3.7
1	yes	5th	1	0	0.5	NPM1	p.Trp288Cysfs*12	Nucleolar multifunctional protein	22.6	3.4
1	yes	5th	1	0	0.5	FLT3-ITD	Not given[1]	Receptor tyrosine kinase	Not given[1]	Not given[1]
2	yes	1st	87	0	12.5	SRSF2	p.Pro95His	Splicing factor	58.3	69.2
2	yes	1st	87	0	12.5	TET2-mut1	p.Thr1554Aspfs*24	DNA methylation related	38.5	42.6
2	yes	1st	87	0	12.5	TET2-mut2	p.Ala1508Leufs*63	DNA methylation related	30.4	40.6
2	yes	1st	87	0	12.5	TP53	p.Val173Met	Tumor suppressor	19.2	35.0
2	yes	1st	87	0	12.5	CBL	p.Arg420Gln	RAS pathway	10.4	10.7
2	yes	1st	87	0	12.5	ASXL1	p.Ser207del	Chromatin modifying	7.5	1.3
2	yes	1st	87	0	12.5	ASXL1	p.Glu635Argfs*15	Chromatin modifying	0.0	1.0
2	yes	2nd	Not done	0	1.7	TET2-mut1	p.Thr1554Aspfs*24	DNA methylation related	40.3	28.0
2	yes	2nd	Not done	0	1.7	TET2-mut2	p.Ala1508Leufs*63	DNA methylation related	39.2	23.8
2	yes	2nd	Not done	0	1.7	CBL	p.Arg420Gln	RAS pathway	5.7	4.1
2	yes	2nd	Not done	0	1.7	PHF6	p.Val617Phe	Tumor suppressor	5.5	3.7
2	yes	2nd	Not done	0	1.7	SRSF2	p.Pro95His	Splicing factor	3.3	1.0
2	yes	2nd	Not done	0	1.7	JAK2	p.Val617Phe	Receptor tyrosine kinase	2.8	2.8
2	yes	2nd	Not done	0	1.7	TP53	p.Val173Met	Tumor suppressor	1.5	0.4
3	no	1st	2.5	0	5.2	EZH2	No protein change	DNA methylation related	56.6	40.6
3	no	1st	2.5	0	5.2	TET2	p.Gly422Glu	DNA methylation related	51.8	50.9
3	no	1st	2.5	0	5.2	ASXL1	p.Glu480Argfs*5	Chromatin modifying	39.8	32.0
3	no	1st	2.5	0	5.2	SETBP1	p.Asp868Asn	DNA replication	5.4	3.2
3	no	1st	2.5	0	5.2	TET2	No protein change	DNA methylation related	2.1	2.1
3	no	1st	2.5	0	5.2	SETBP1	p.Gly870Ser	DNA replication	1.1	0.0

4	yes	1st	65	72	4.2	IDH1	p.Arg132Cys	DNA methylation related	92.5	87.9
4	yes	1st	65	72	4.2	NPM1	p.Trp288Cysfs*12	Nucleolar multifunctional protein	49.2	45.9
4	yes	1st	65	72	4.2	GATA2	p.Arg362Gln	Transcription factor	45.3	44.1
4	yes	2nd	19	2	1.7	IDH1	p.Arg132Cys	DNA methylation related	69.7	47.3
4	yes	2nd	19	2	1.7	NPM1	p.Trp288Cysfs*12	Nucleolar multifunctional protein	40.3	32.0
4	yes	2nd	19	2	1.7	GATA2	p.Arg362Gln	Transcription factor	37.8	28.3
4	yes	2nd	19	2	1.7	NRAS	p.Gly12Asp	RAS pathway	1.3	0.0
4	yes	2nd	19	2	1.7	DNMT3A	No protein change	DNA methylation related	1.0	2.0
5	yes	1st	3	0	3.1	TET2	p.Gln1510*	DNA methylation related	8.9	3.7
5	yes	2nd	3	Not done	1.5	RB1	p.Arg358*	Tumor suppressor	1.9	0.0
5	yes	2nd	3	Not done	1.5	TET2	p.Gln1510*	DNA methylation related	1.5	2.7
6	no	1st	2.5	2	3.7	ASXL1	p.Gly646Trpfs*12	Chromatin modifying	40.5	39.4
6	no	1st	2.5	2	3.7	U2AF1	p.Arg156His	Splicing factor	39.8	40.8
6	no	1st	2.5	2	3.7	SRSF2	p.Pro95Arg	Splicing factor	2.2	0.0
6	no	1st	2.5	2	3.7	DNMT3A	p.Ile670Hisfs*43	DNA methylation related	0.3	1.3
7	no	1st	8	0	2.5	ASXL1	p.Arg693*	Chromatin modifying	2.5	0.0
8	no	1st	40	0	3.1	TET2-mut1	p.Pro1528_Gln1540del	DNA methylation related	61.4	58.5
8	no	1st	40	0	3.1	TET2-mut2	p.Gln821*	DNA methylation related	47.2	37.8
8	no	1st	40	0	3.1	SRSF2	p.Arg94dup	Splicing factor	45.9	35.1
8	no	1st	40	0	3.1	DNMT3A	No protein change	DNA methylation related	42.7	35.6
8	no	1st	40	0	3.1	TET2-mut3	p.Gln1466Pro	DNA methylation related	33.6	20.8
8	no	1st	40	0	3.1	TET2-mut4	p.Gln1466Leufs*12	DNA methylation related	33.5	21.0
8	no	1st	40	0	3.1	RUNX1-mut1	p.Leu98Serfs*24	Transcription factor	32.2	19.0
8	no	1st	40	0	3.1	RUNX1-mut2	p.Pro203Leufs*8	Transcription factor	13.0	15.3
8	no	1st	40	0	3.1	RUNX1-mut3	p.Gly165Alafs*14	Transcription factor	3.7	0.0
9	no	1st	2.5	0	6.0	IDH1	p.Arg132Cys	DNA methylation related	9.1	0.0

ID indicates patient identification number; BM, bone marrow; PB, peripheral blood; WBC, white blood cell count; HGVSp, Human genome Variation Society; VAF, variant allele frequency; ITD, internal tandem duplication; DNA, deoxyribonucleic acid.

[1]FLT3-ITD mutations are not included in the diagram as the method used for detection does not produce VAF values.

Figure S7. AML patient with serial analyses and a discordant NPM1 mutation in the third analysis[1].

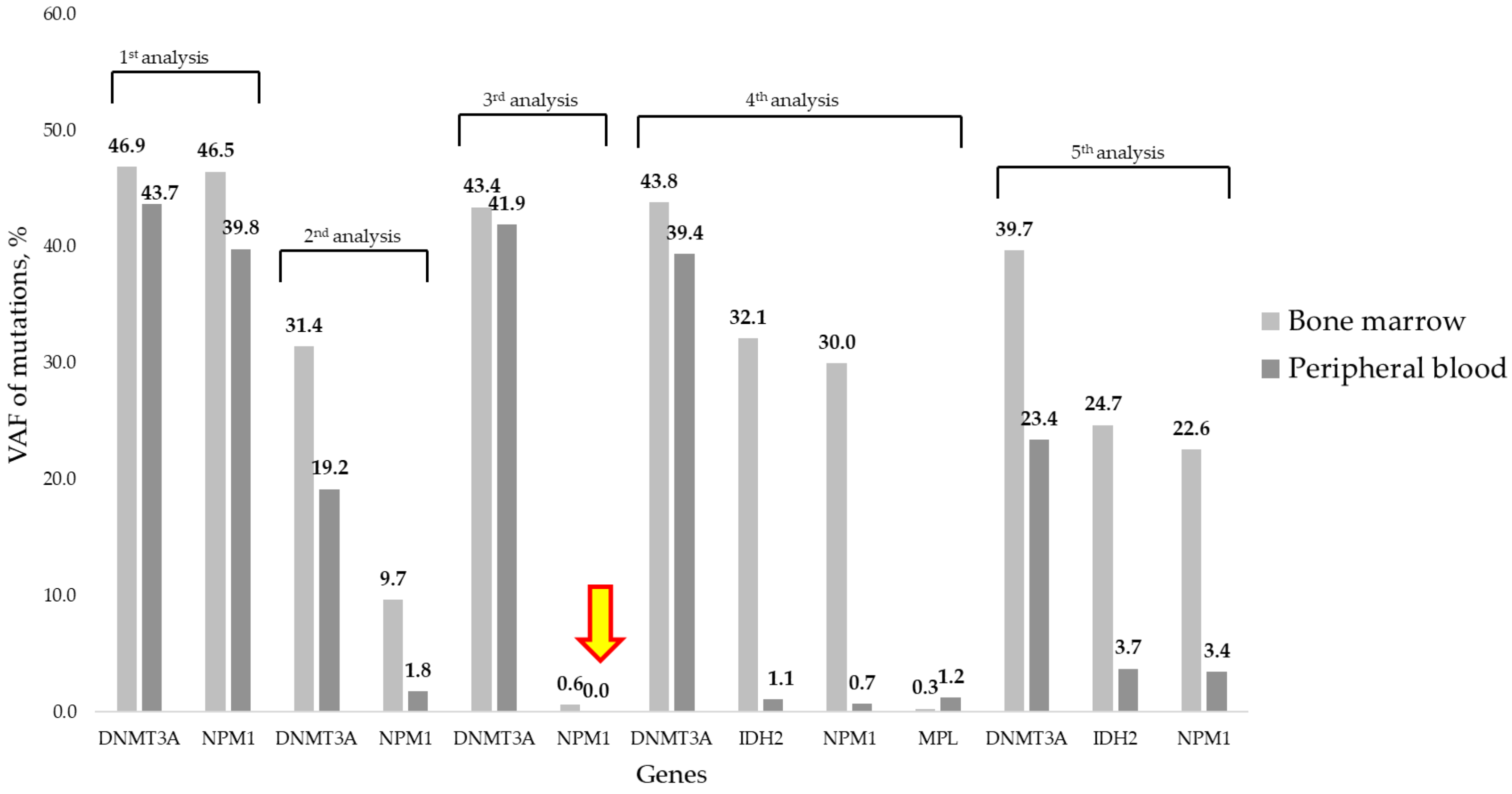

The red arrow denotes the discordant mutation.

Patient information: Age 55, 1st analysis: 8% peripheral blood blasts, 95% blasts in bone marrow aspirate, 95% blasts in bone marrow histology, white blood cell count 32.7 G/L; 2nd analysis: 2% blasts in bone marrow aspirate, white blood cell count (G/L) 0.7; 3rd analysis: 0% blasts in peripheral blood count, 1% blasts in bone marrow aspirate, white blood cell count (G/L) 4.6; 4th analysis: 0% blasts in peripheral blood count, 78% blasts in bone marrow aspirate, white blood cell count (G/L) 1.1; 5th analysis: 0% blasts in peripheral blood count, 1% blasts in bone marrow aspirate, white blood cell count (G/L) 0.5.

[1]FLT3 ITD mutations in 1st, 4th and 5th analysis are not shown in the graph.

Figure S8. AML patient with serial analyses and a discordant ASXL1 mutation in the first analysis.

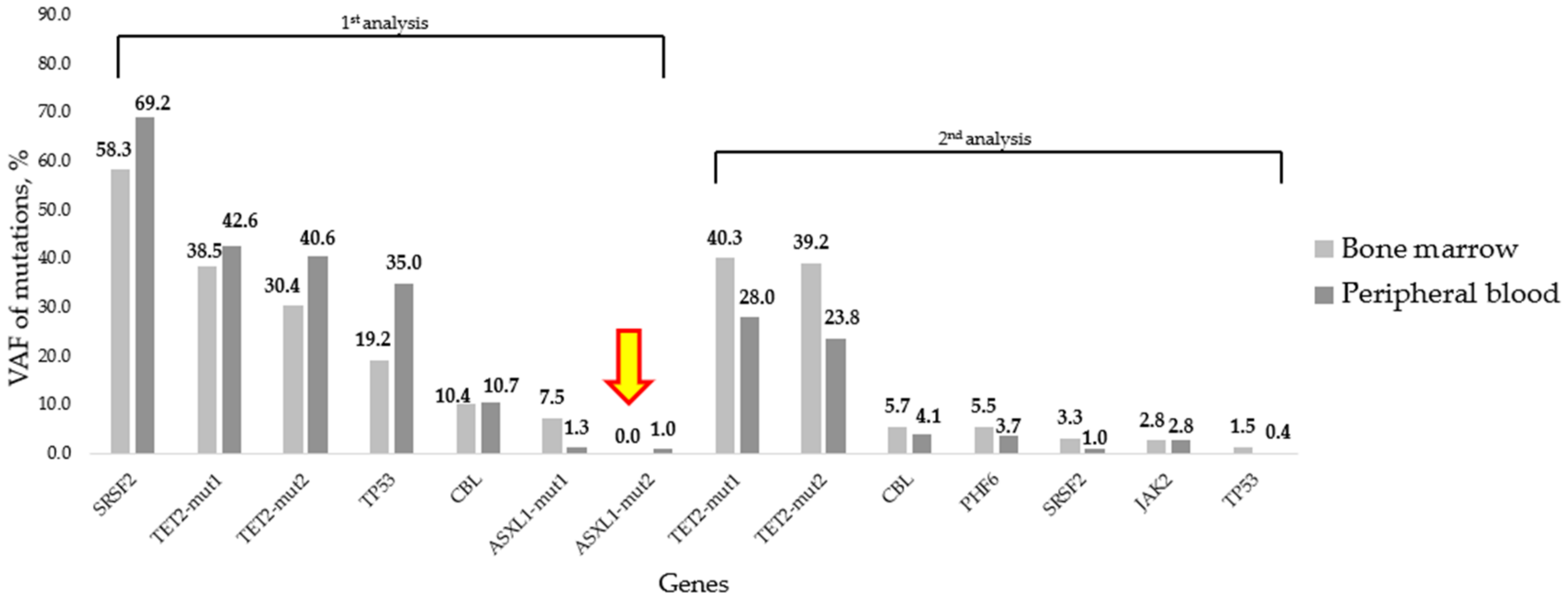

The red arrow denotes the discordant mutation.

Patient information: Age 84, 1st analysis: 1% peripheral blood blasts, 87% blasts in bone marrow aspirate, white blood cell count 12.5 G/L; 2nd analysis: 0 % peripheral blood blasts, white blood cell count 1.7 G/L.

Figure S9. MDS patient with a discordant SETBP1 mutation.

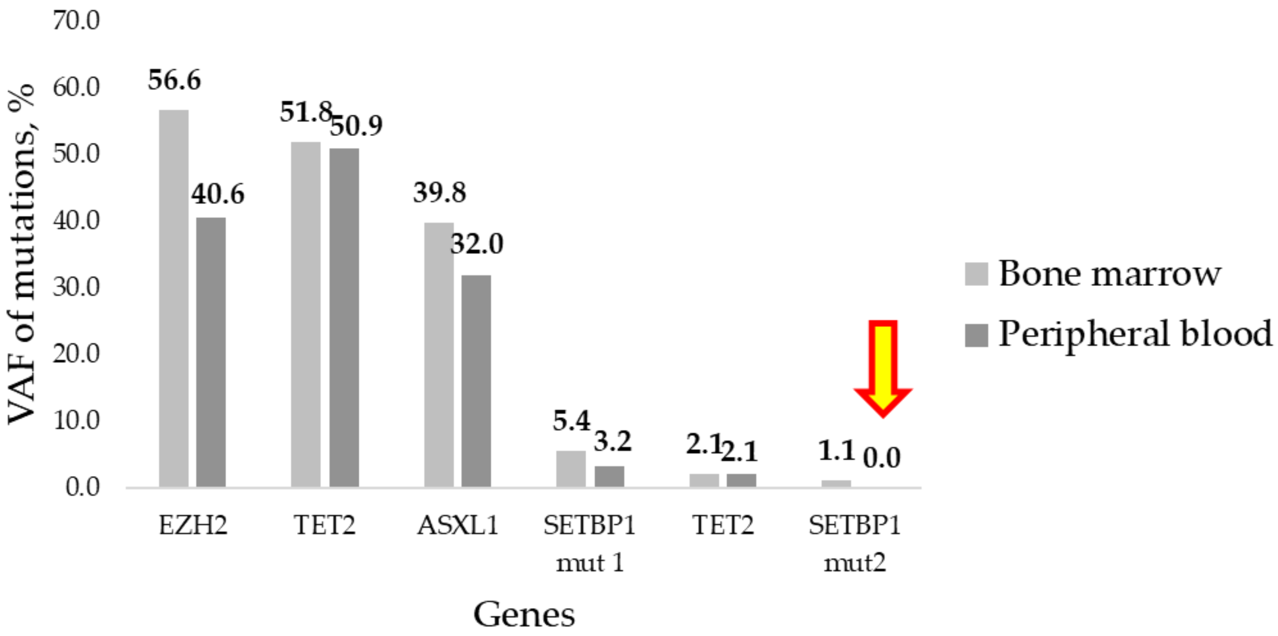

The red arrow denotes the discordant mutation.

Patient information: Age 83, 0% peripheral blood blasts, 2% blasts in bone marrow aspirate, 2.5% blasts in bone marrow histology, white blood cell count 5.2 G/L.

Figure S10. AML patient with serial analyses and a discordant NRAS mutation in the second analysis.

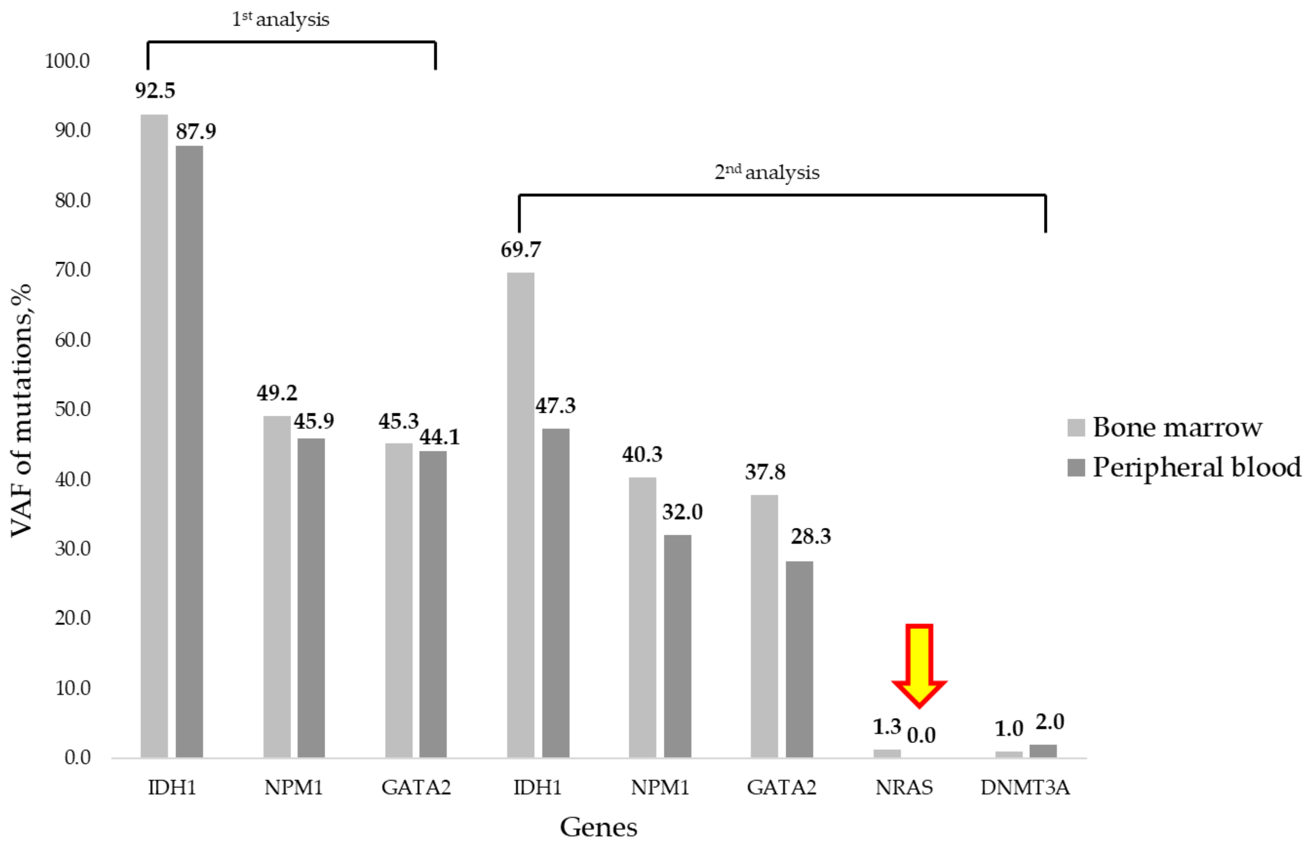

The red arrow denotes the discordant mutation.

Patient information: Age 81, 1st analysis: 72% peripheral blood blasts, 63% blasts in bone marrow aspirate, 65% blasts in bone marrow histology, white blood cell count 4.2 G/L; 2nd analysis: 2% peripheral blood blasts, 15% blasts in bone marrow aspirate, 19% blasts in bone marrow histology, white blood cell count 1.7 G/L.

Figure S11. MDS patient with serial analyses and a discordant RB1 mutation in the second analysis.

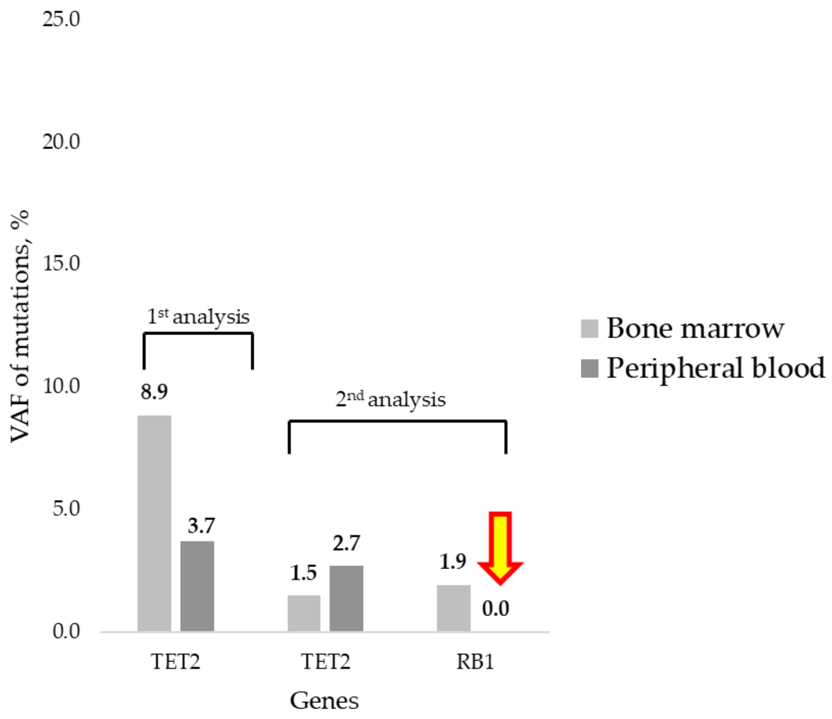

The red arrow denotes the discordant mutation.

Patient information: Age 79,1st analysis 0% peripheral blood blasts, 3% blasts in bone marrow aspirate, 2.5% blasts in bone marrow histology, white blood cell count 3.1 G/L; 2nd analysis: 3% blasts in bone marrow aspirate, 2.5% blasts in bone marrow histology, white blood cell count 1.5 G/L.

Figure S12. MPN patient with a discordant SRSF2 mutation.

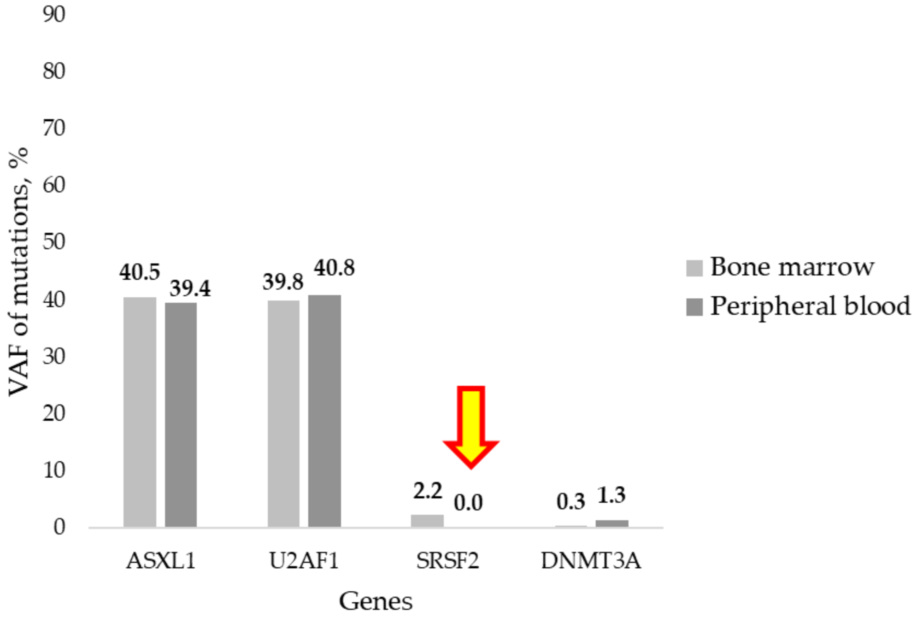

The red arrow denotes the discordant mutation.

Patient information: Age 75, 2% peripheral blood blasts, 2% blasts in bone marrow aspirate, 2.5% blasts in bone marrow histology, white blood cell count 3.7 G/L.

Figure S13. MDS patient with a discordant ASXL1 mutation.

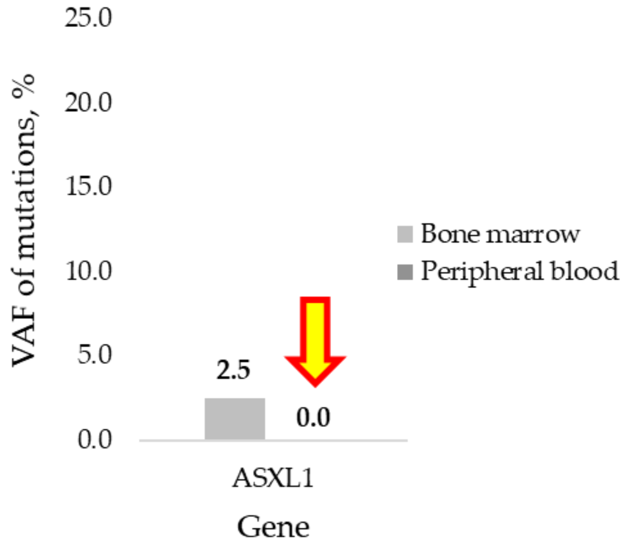

The red arrow denotes the discordant mutation.

Patient information: Age 70, 0% peripheral blood blasts, 8 % blasts in bone marrow aspirate, 7.5% blasts in bone marrow histology, white blood cell count 2.5 G/L.

Figure S14. AML patient with a discordant RUNX1 mutation.

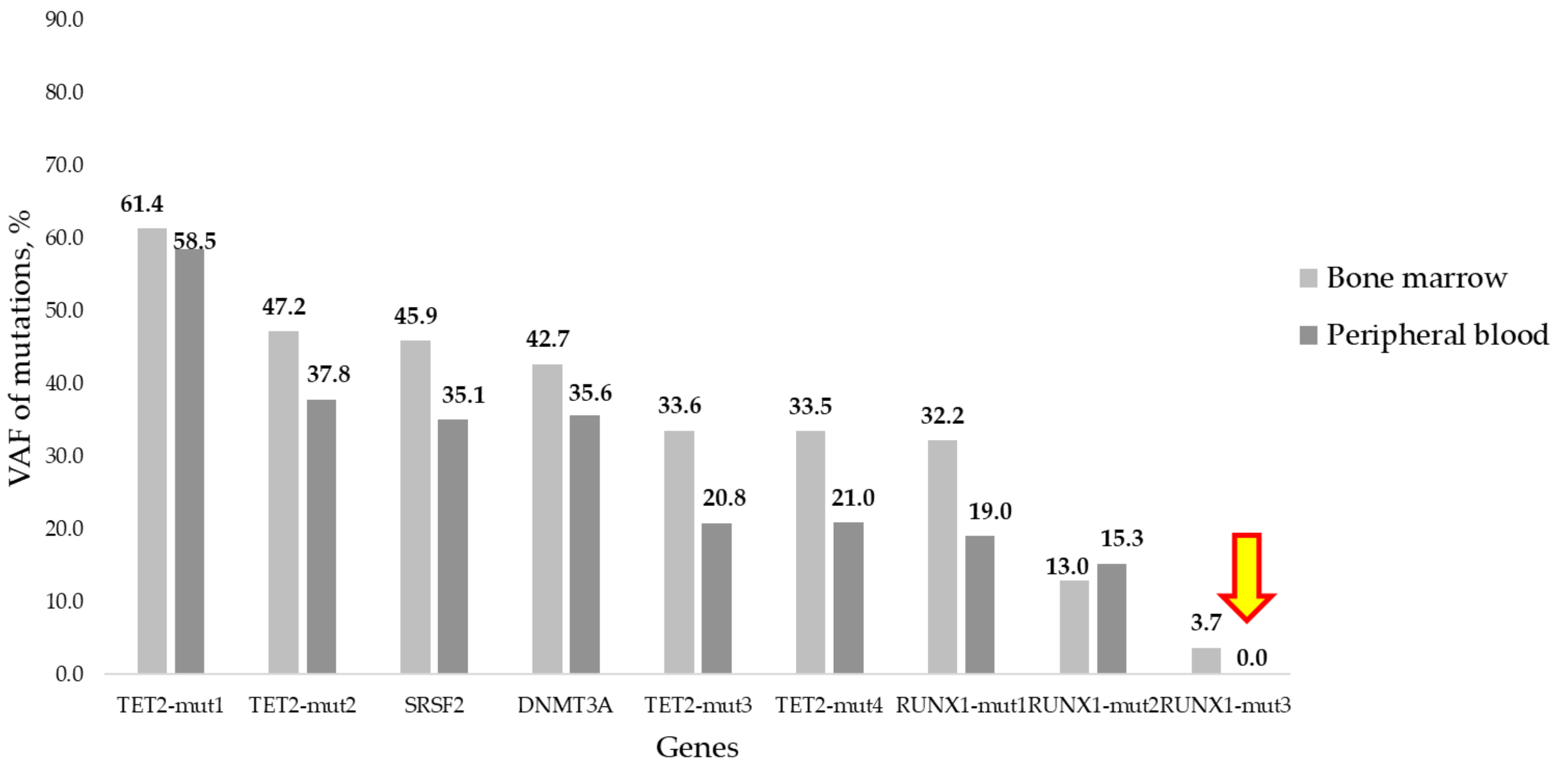

The red arrow denotes the discordant mutation.

Patient information: Age 76, 0% peripheral blood blasts, 5% blasts in bone marrow aspirate, 40% blasts in bone marrow histology, white blood cell count 3.1 G/L.

Figure S15. Waldenstrom's disease patient with a discordant IDH1 mutation.

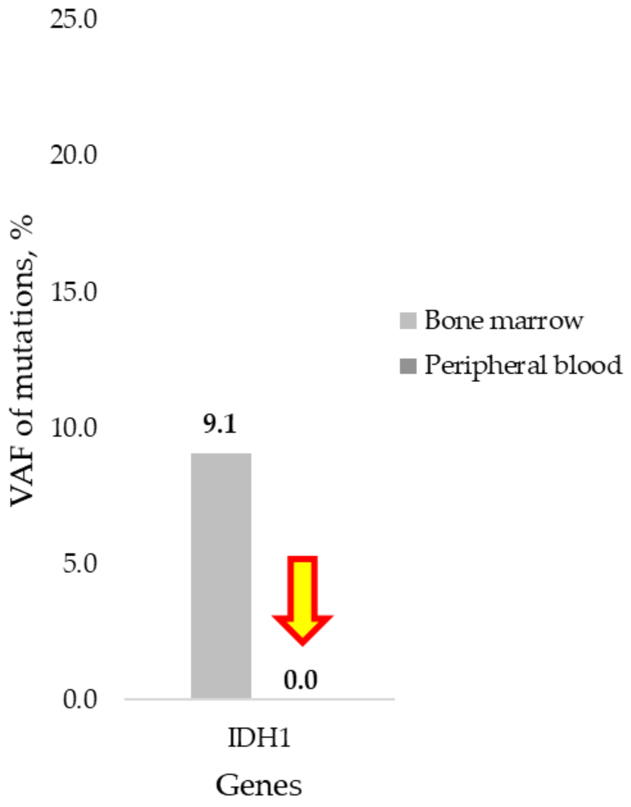

The red arrow denotes the discordant mutation.

Patient information: Age 70, 0% peripheral blood blasts, 1% blasts in bone marrow aspirate, 2.5% blasts in bone marrow histology, white blood cell count 6.0 G/L.

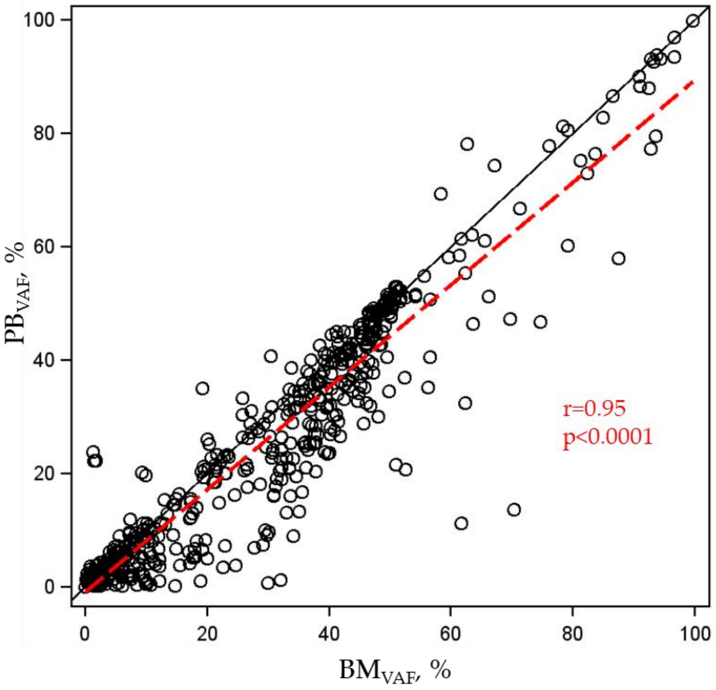

Figure S16. Scatterplot of the variant allele frequency (VAF) of the mutated genes in bone marrow and peripheral blood of samples drawn on the same day. The black line is the bisecting line showing a perfect linear regression with slope = 1 and intercept = 0. The red dashed line is the regression line, indicating the correlation between BM$_{VAF}$ and PB$_{VAF}$ of each paired sample.

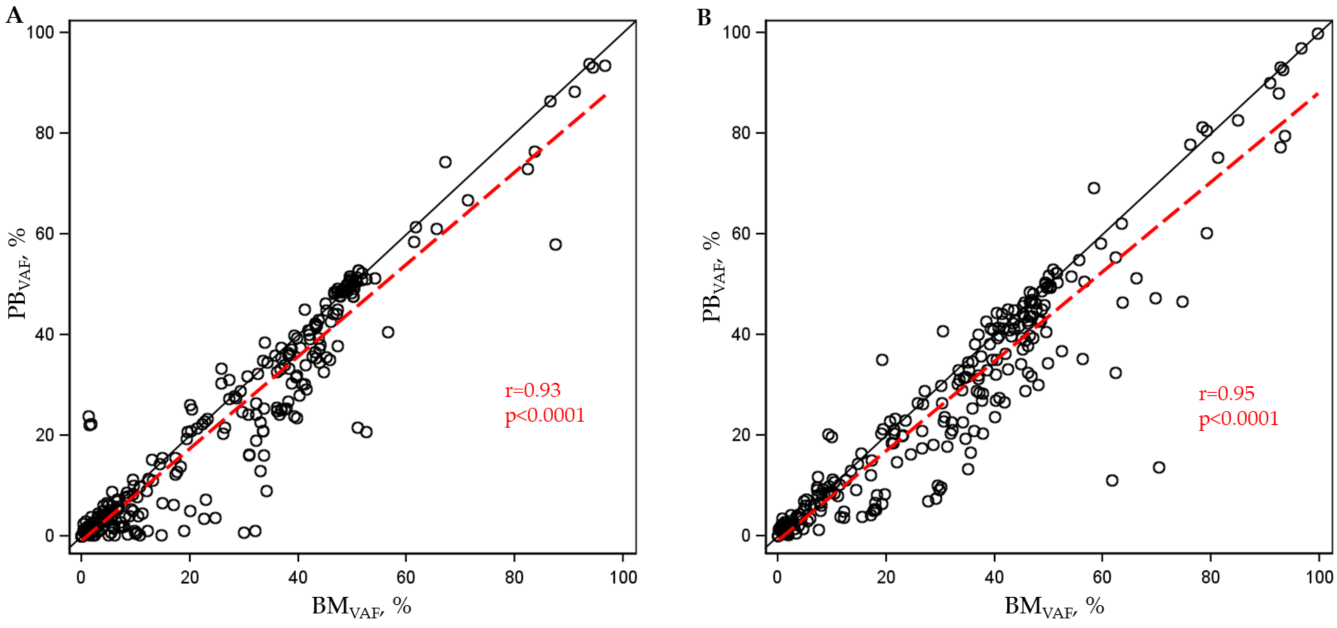

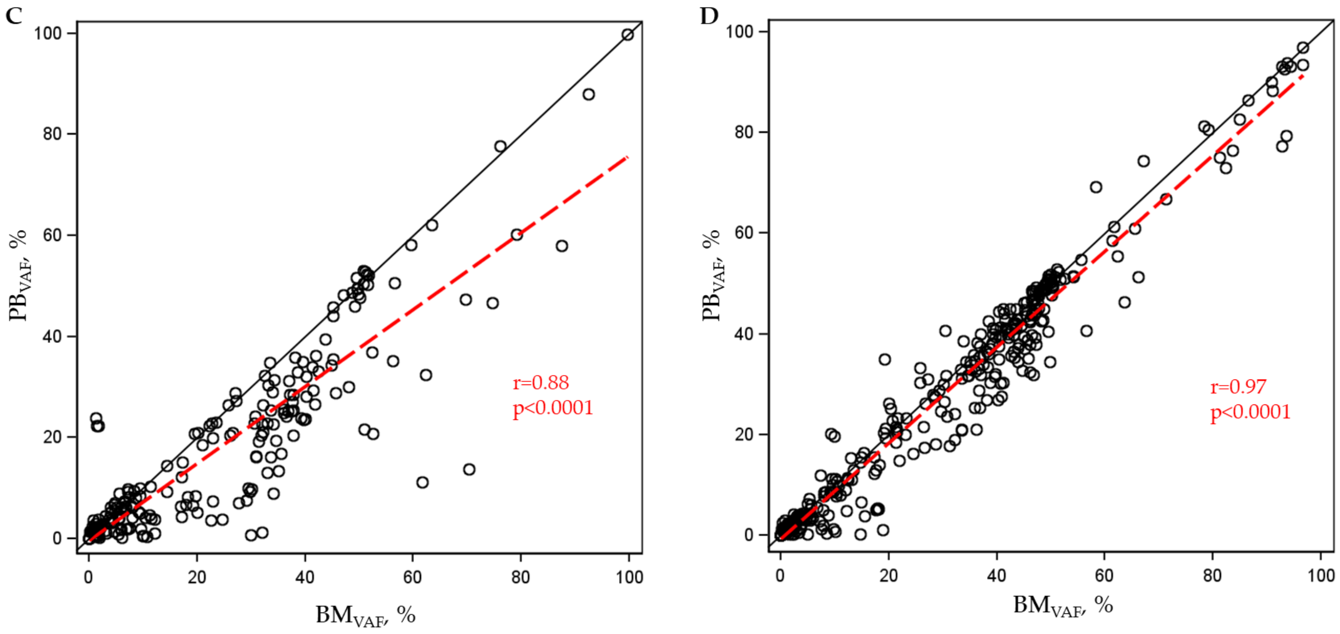

Figure S17. Scatterplot of the variant allele frequency (VAF) of the mutated genes in bone marrow and peripheral blood of samples drawn on the same day. For patient samples with the following peripheral blood parameters on the day of bone marrow sampling **(A)** Peripheral blast percentage =0%. **(B)** Peripheral blast percentage ≥1%. **(C)** Absolute neutrophil count <1.0 10^9/L. **(D)** Absolute neutrophil count ≥1.0 10^9/L. The black line is the bisecting line showing a perfect linear regression with slope = 1 and intercept = 0. The red dashed line is the regression line, indicating the correlation between BM_{VAF} and PB_{VAF} of each paired sample.

A

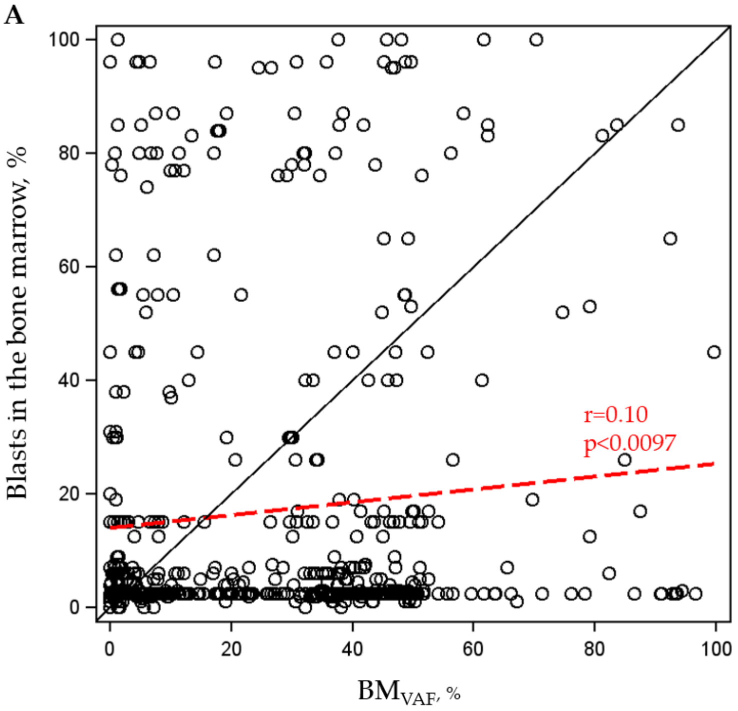

B

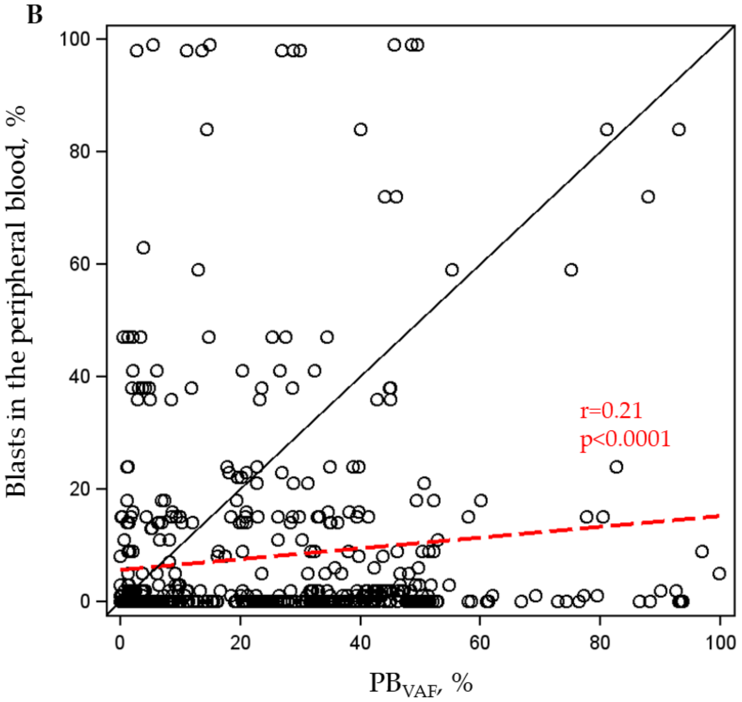

Figure S18. Scatterplots of the "variant allele frequency (VAF) of a mutation" vs "blast percentage" of samples drawn on the same day. (A) "BM_{VAF}" vs "percentage of bone marrow blasts". **(B)** "PB_{VAF}" vs "percentage of peripheral blood blasts". The black line is the bisecting line showing a perfect linear regression with slope = 1 and intercept = 0. The red dashed line is the regression line, indicating the correlation between BM_{VAF} and PB_{VAF} of each paired sample.

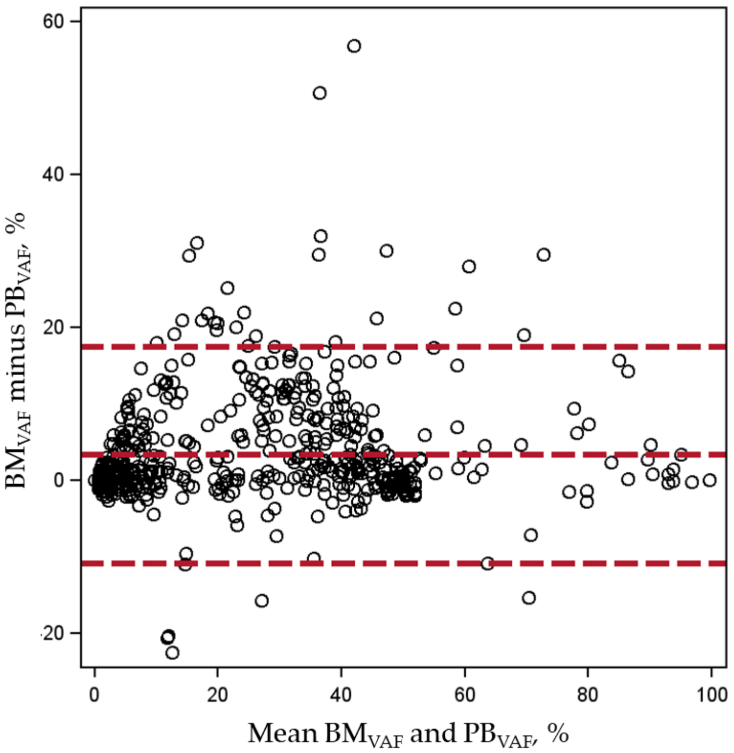

Figure S19. Scatterplot of the variant allele frequency (VAF): difference against the mean of samples drawn on the same day [1] The X-axis shows the mean of the BM_{VAF} and the PB_{VAF}. The Y-axis displays the difference between the BM_{VAF} and the PB_{VAF}. The central red dashed line represents the mean of the difference, and the outer red dashed lines correspond to $\bar{d} \pm 2\bar{\sigma}$, where $\bar{\sigma}$ represents the standard deviation of the difference. As discussed in [55], the region between these two outer lines is referred to as the "Limits of Agreement" and it visualizes the difference between the two methods of measurement. [1]Analysed according to Bland et al, Lancet 1986 [55].

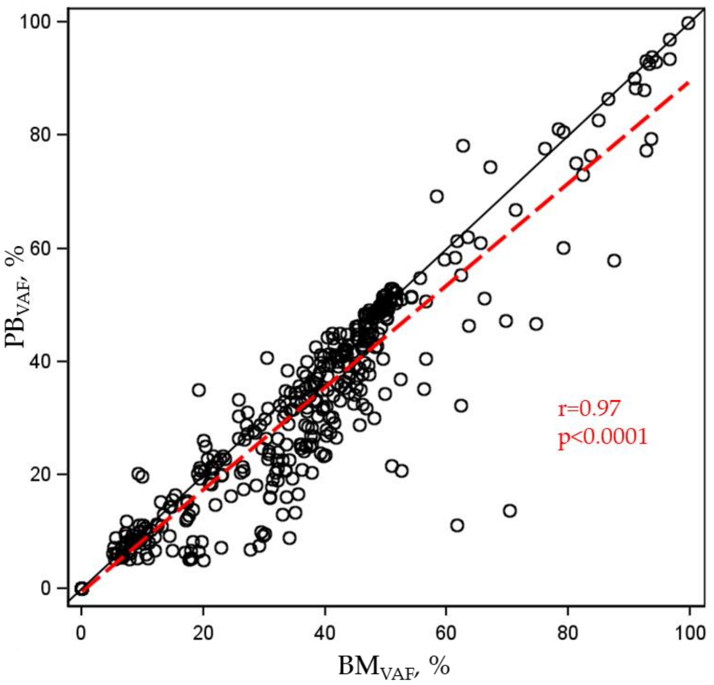

Figure S20. Scatterplot of the variant allele frequency (VAF) of the mutated genes in bone marrow and peripheral blood of samples drawn on the same day and mutations detected with a VAF >5%. The black line is the bisecting line showing a perfect linear regression with slope = 1 and intercept = 0. The red dashed line is the regression line, indicating the correlation between BM_{VAF} and PB_{VAF} of each paired sample.

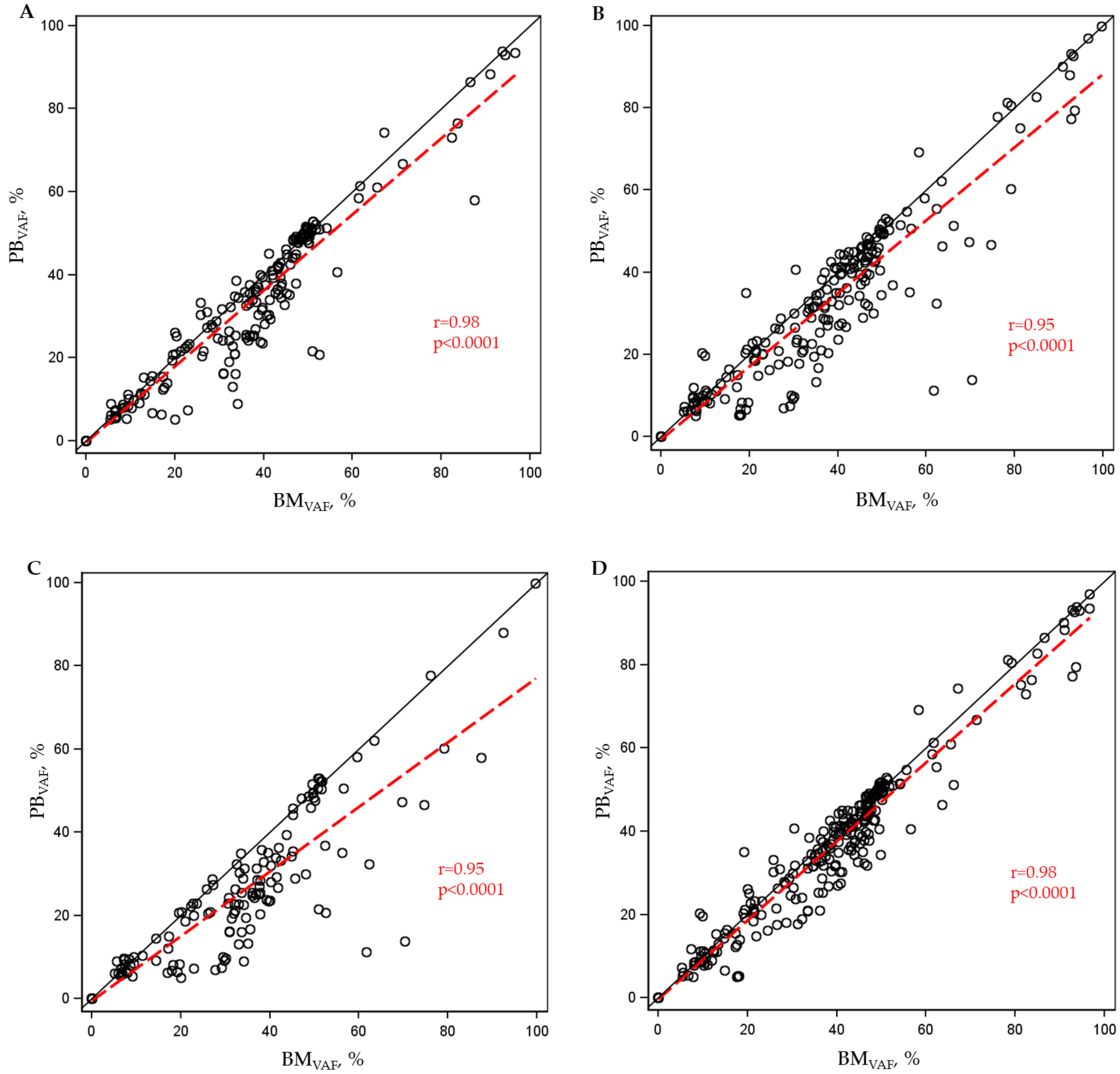

Figure S21. Scatterplot of the variant allele frequency (VAF) of the mutated genes in bone marrow and peripheral blood of samples drawn on the same day and mutations detected with a VAF >5%. For patient samples with the following peripheral blood parameters on the day of bone marrow sampling **(A)** Peripheral blast percentage =0%. **(B)** Peripheral blast percentage ≥1%. **(C)** Absolute neutrophil count <1.0 10^9/L. **(D)** Absolute neutrophil count ≥1.0 10^9/L. The black line is the bisecting line showing a perfect linear regression with slope = 1 and intercept = 0. The red dashed line is the regression line, indicating the correlation between BM$_{VAF}$ and PB$_{VAF}$ of each paired sample.

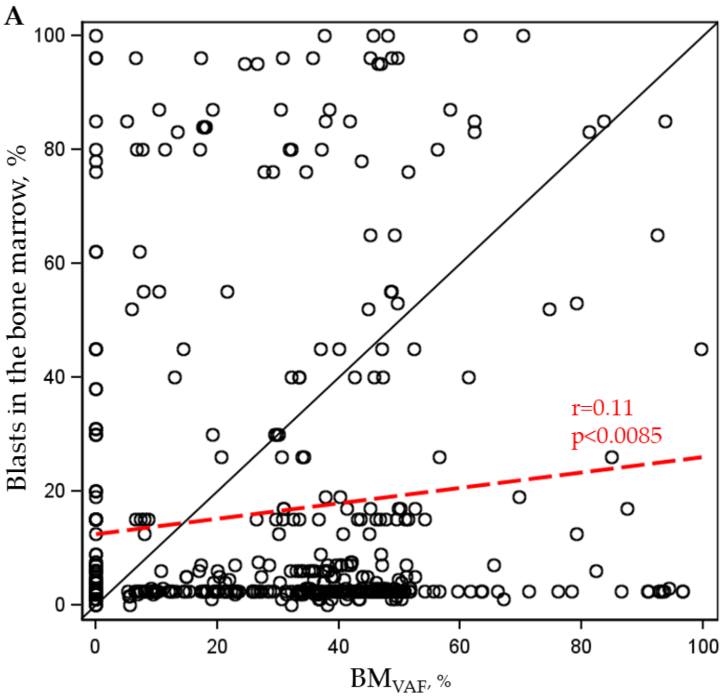

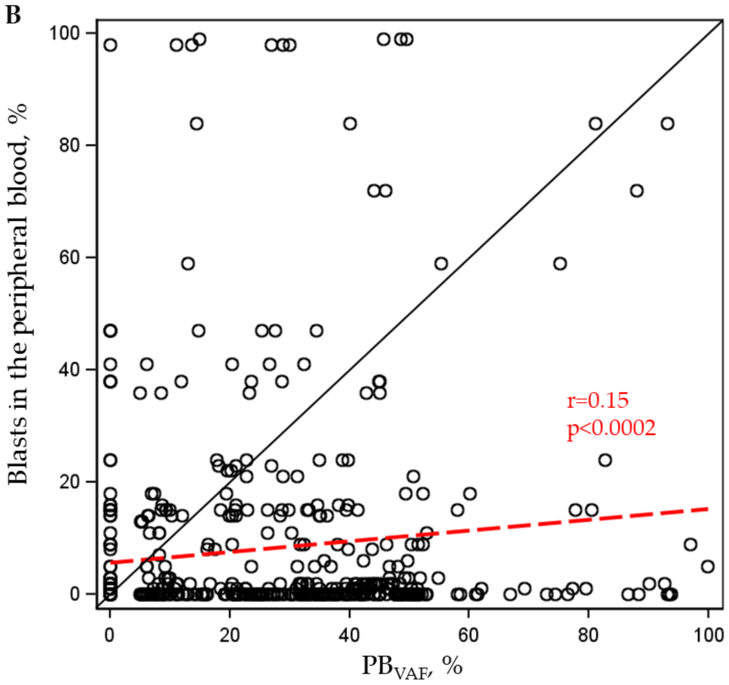

Figure S22. Scatterplots of the "variant allele frequency (VAF) of a mutation" vs "blast percentage" of samples drawn on the same day and mutations detected with a VAF >5%. (A) "BM_{VAF}" vs "percentage of bone marrow blasts". **(B)** "PB_{VAF}" vs "percentage of peripheral blood blasts". The black line is the bisecting line showing a perfect linear regression with slope = 1 and intercept = 0. The red dashed line is the regression line, indicating the correlation between BM_{VAF} and PB_{VAF} of each paired sample.

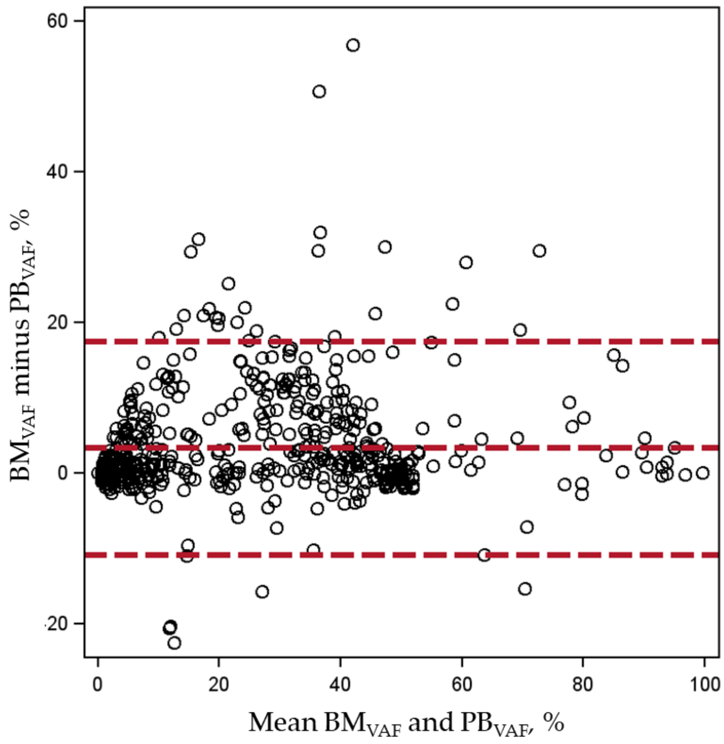

Figure S23. Scatterplot of the variant allele frequency (VAF): difference against the mean of samples drawn on the same day and mutations detected with a VAF >5%. [1] The X-axis shows the mean of the BM_{VAF} and the PB_{VAF}. The Y-axis displays the difference between the BM_{VAF} and the PB_{VAF}. The central red dashed line represents the mean of the difference, and the outer red dashed lines correspond to $\bar{d} \pm 2\bar{\sigma}$, where $\bar{\sigma}$ represents the standard deviation of the difference. As discussed in [55], the region between these two outer lines is referred to as the "Limits of Agreement" and it visualizes the difference between the two methods of measurement. [1]Analysed according to Bland et al, Lancet 1986 [55].

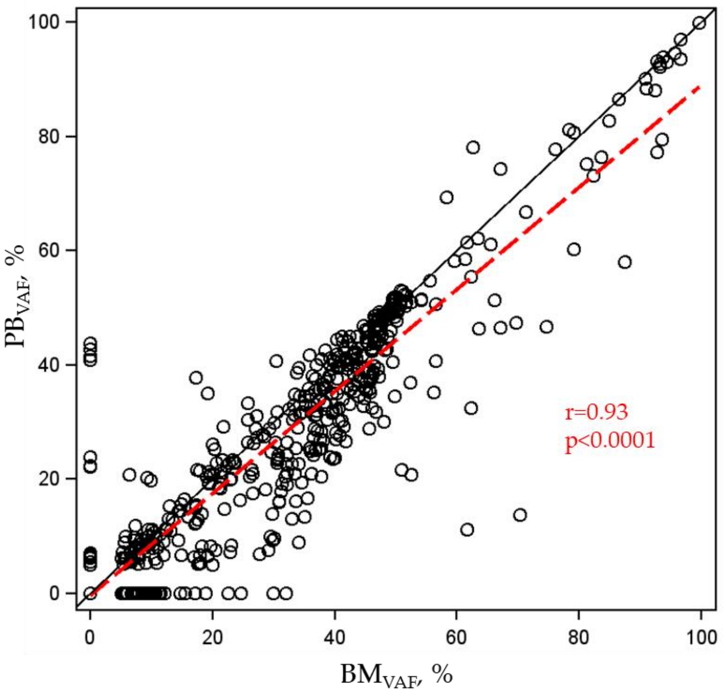

Figure S24. Scatterplot of the variant allele frequency (VAF) of the mutated genes in bone marrow and peripheral blood of samples with mutations >5% VAF. The black line is the bisecting line showing a perfect linear regression with slope = 1 and intercept = 0. The red dashed line is the regression line, indicating the correlation between BM_{VAF} and PB_{VAF} of each paired sample.

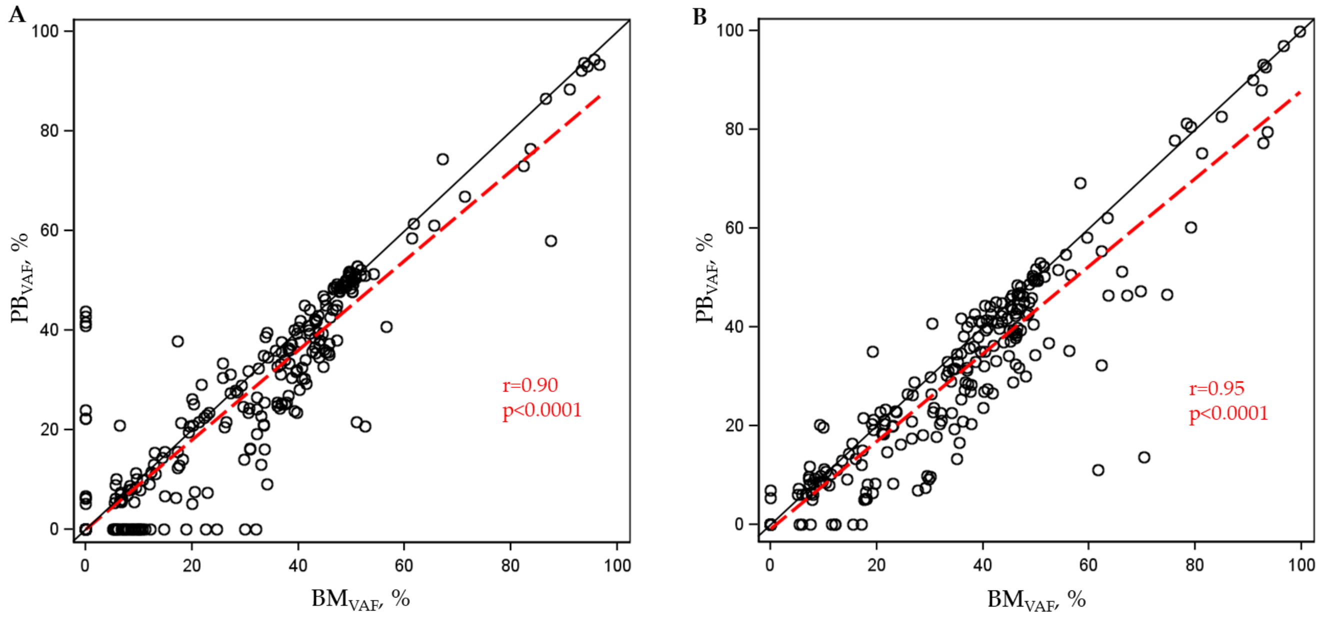

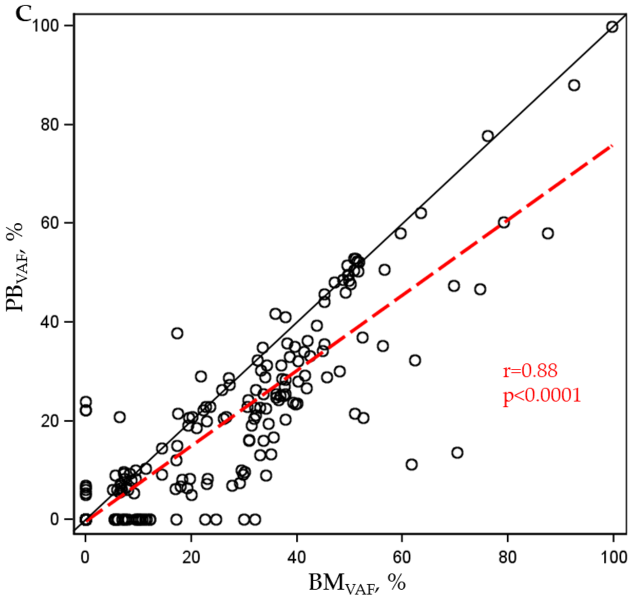

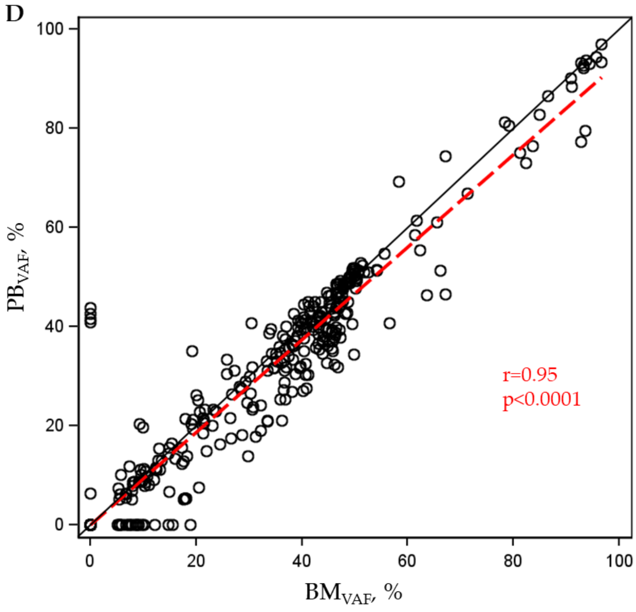

Figure S25. Scatterplot of the variant allele frequency (VAF) of the mutated genes in bone marrow and peripheral blood of samples with mutations >5% VAF. For patient samples with the following peripheral blood parameters on the day of bone marrow sampling **(A)** Peripheral blast percentage =0%. **(B)** Peripheral blast percentage ≥1%. **(C)** Absolute neutrophil count <1.0 10^9/L. **(D)** Absolute neutrophil count ≥1.0 10^9/L. The black line is the bisecting line showing a perfect linear regression with slope = 1 and intercept = 0. The red dashed line is the regression line, indicating the correlation between BM_{VAF} and PB_{VAF} of each paired sample.

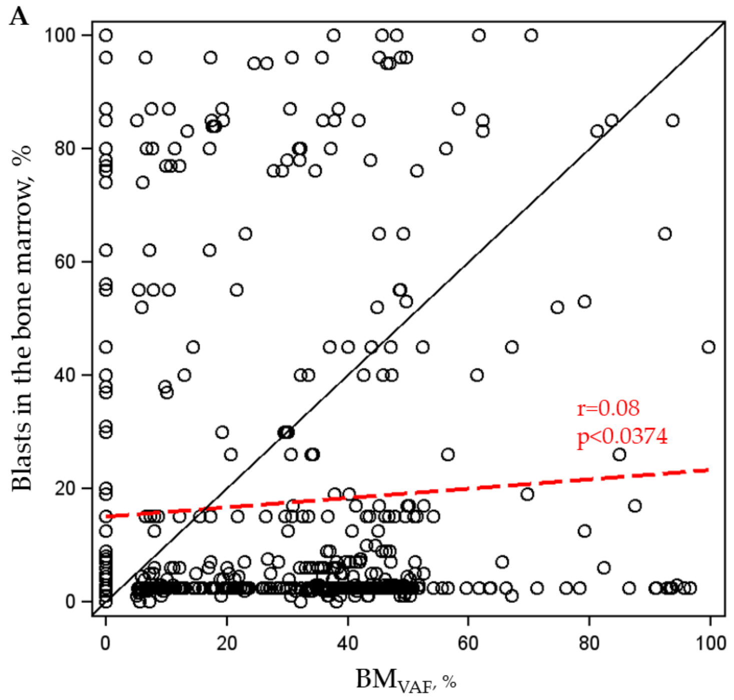

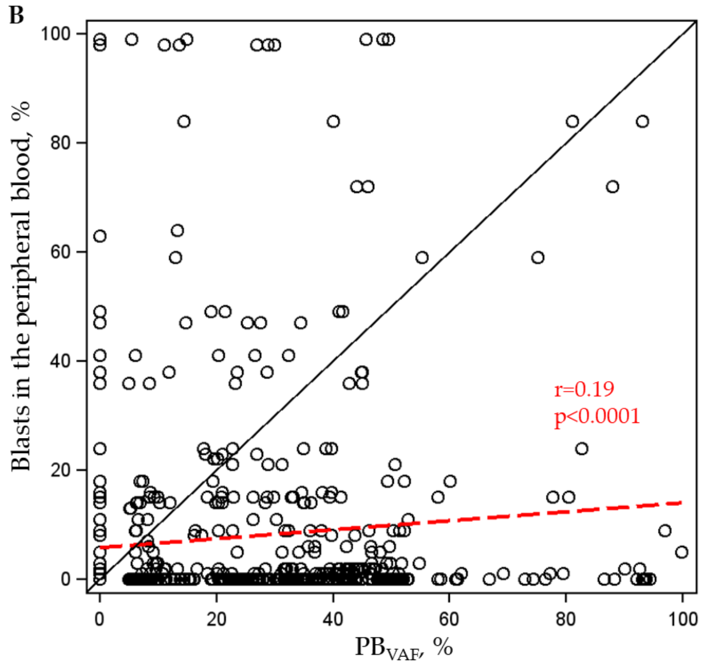

Figure S26. Scatterplots of the "variant allele frequency (VAF) of a mutation" vs "blast percentage" of samples with mutations >5% VAF. (A) "BM_{VAF}" vs "percentage of bone marrow blasts". **(B)** "PB_{VAF}" vs "percentage of peripheral blood blasts". The black line is the bisecting line showing a perfect linear regression with slope = 1 and intercept = 0. The red dashed line is the regression line, indicating the correlation between BM_{VAF} and PB_{VAF} of each paired sample.

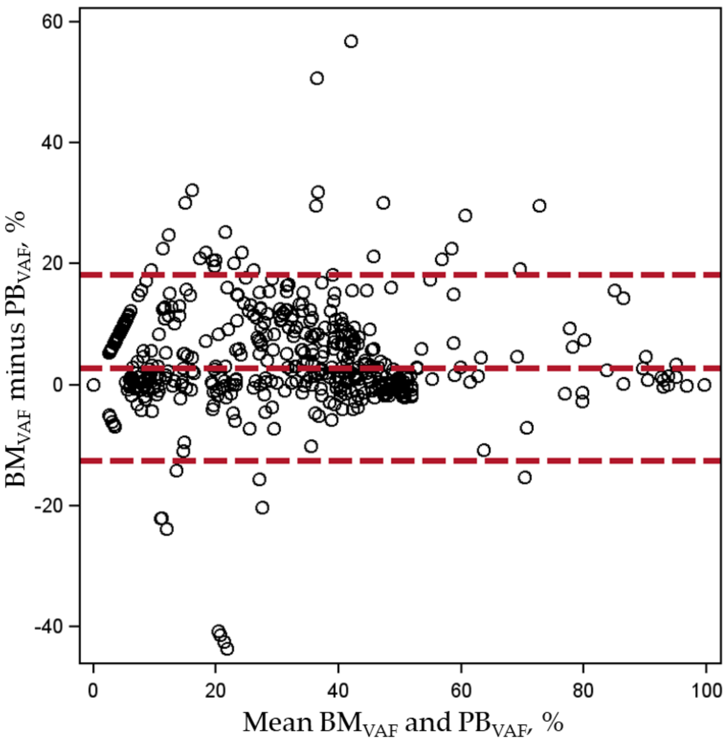

Figure S27. Scatterplot of the variant allele frequency (VAF): difference against the mean of samples with mutations >5% VAF.[1] The X-axis shows the mean of the BM_{VAF} and the PB_{VAF}. The Y-axis displays the difference between the BM_{VAF} and the PB_{VAF}. The central red dashed line represents the mean of the difference, and the outer red dashed lines correspond to $\bar{d} \pm 2\bar{\sigma}$, where $\bar{\sigma}$ represents the standard deviation of the difference. As discussed in [55], the region between these two outer lines is referred to as the "Limits of Agreement" and it visualizes the difference between the two methods of measurement. [1]Analysed according to Bland et al, Lancet 1986 [55].
